# Precise Ratiometric Drug Delivery for the Treatment of Triple-Negative Breast Cancer

**DOI:** 10.1021/acsnano.5c13083

**Published:** 2025-11-19

**Authors:** Rae Hyung Kang, Morteza Rasoulianboroujeni, Maryam Kianpour, Lauren Repp, Suzanne M. Ponik, Glen S. Kwon

**Affiliations:** † Pharmaceutical Sciences Division, School of Pharmacy, 5228 University of Wisconsin-Madison, Madison, Wisconsin 53705, United States; ‡ Department of Pharmaceutical Engineering, Dankook University, Cheonan 31116, Republic of Korea; § Department of Pharmaceutical Sciences, Gatton College of Pharmacy, East Tennessee State University, Johnson City, Tennessee 37614, United States; ∥ Department of Cell and Regenerative Biology, School of Medicine and Public Health, University of Wisconsin-Madison, Madison, Wisconsin 53705, United States

**Keywords:** paclitaxel, rapamycin, polymeric micelle, ratiometric drug delivery, triple-negative breast cancer

## Abstract

Triple-negative breast cancer (TNBC) remains a significant clinical challenge due to its high aggressiveness, poor prognosis, and lack of targeted therapies. Combining paclitaxel (PTX) with rapamycin (RAP), a PI3K/AKT/mTOR pathway inhibitor, has shown promise in preclinical and clinical studies, but the approach is limited by pharmacokinetic disparities and toxicity concerns. Here, we introduce Rapaxane, a formulation composed of polymeric micelles coloaded with oligo­(lactic acid)_8_ conjugated prodrugs of PTX (oLA_8_-PTX) and RAP (oLA_8_-RAP) at an optimized synergistic ratio (5:1). We evaluated the efficacy of Rapaxane in vitro and in preclinical TNBC models, comparing it to the benchmark formulation Abraxane, the combination of parent drugs, and monotherapies using the prodrugs. In vitro, Rapaxane demonstrated notable cytotoxicity against the 4T1 and MDA-MB-231 TNBC cell lines. Ratiometric encapsulation, stability, synchronized drug release, and conversion were confirmed using dynamic light scattering (DLS) and reverse-phase high-performance liquid chromatography (RP-HPLC). Hemolysis assays indicated negligible toxicity, confirming the safety of Rapaxane for intravenous administration. In vivo, Rapaxane significantly reduced tumor growth and metastasis while improving survival rates in subcutaneous and orthotopic TNBC mouse models. Histological analysis using H&E staining, complemented by Ki-67 immunohistochemical staining, demonstrated effective inhibition of lung metastasis in Rapaxane-treated groups compared to control groups. Rapaxane’s ability to maintain precise ratiometric dosing, sustain drug release, and enhance therapeutic efficacy while mitigating adverse effects underscores its potential as a next-generation therapy for TNBC. This study highlights the feasibility of nanotechnology-based ratiometric drug delivery systems in overcoming the limitations of conventional combination therapies, paving the way for more effective treatment options for aggressive cancers like TNBC.

## Graphical abstract

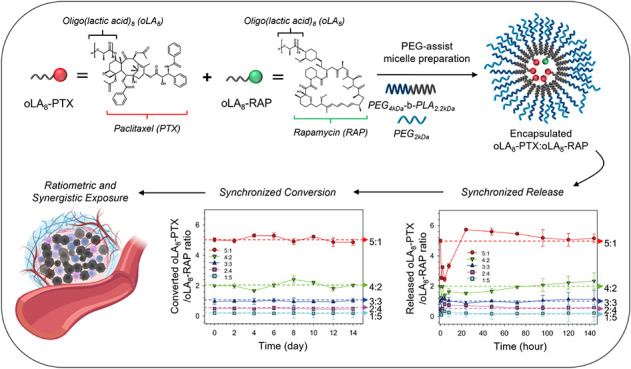

## Introduction

Triple Negative Breast Cancer (TNBC), accounting for 15% of all BCs, remains the least responsive subgroup to treatment.^1–3^ Shorter progression-free survival (PFS) and overall survival (OS) in TNBC can be attributed to poor prognosis and high aggressiveness of the disease.^4^ TNBC patients also exhibit an increased predisposition to developing metastases, leading to adverse clinical outcomes when compared to other subgroups.^5,6^

Even though the management of TNBC lacks solid guidelines, the National Comprehensive Cancer Network (NCCN), European Society for Medical Oncology (ESMO), and Associazione Italiana di Oncologia Medica (AIOM) recommend the use of taxanes, such as paclitaxel (PTX) in mono or combination therapy,^7^ and this group of chemotherapeutics is frequently listed as first-line treatment options against TNBC despite their short-lasting benefits.^8^ PTX causes tumor cell death by blocking the normal cell cycle through binding to intracellular β-tubulin to stabilize microtubules.^9^ However, PTX has also been reported to modulate proliferation and cell survival pathways, such as the mitogen-activated protein kinase kinase (MEK), extracellular signal-regulated kinase (ERK) signaling cascade, and phosphatidylinositol 3′-kinase (PI3K)/Akt/mTOR pathway.^9–14^ The PI3K/AKT/mTOR pathway serves as the primary signaling pathway governing the regulation of cell proliferation, survival, metabolism, and motility, and is frequently activated in breast cancer^15–17^ with the highest activation in TNBC compared to other subtypes.^6,18,19^ It has been shown that PI3K/AKT/mTOR signaling is often altered in TNBC patients.^20^ Activation of this signaling pathway has been suggested to confer resistance to chemotherapy in TNBC.^21^ Therefore, the concurrent administration of PTX with a PI3K/AKT/mTOR signaling pathway inhibitor has emerged as a promising therapeutic approach for treating TNBC. In a phase II clinical trial, Schmid et al.^22^ assessed the safety and efficacy of adding capivasertib, an oral AKT inhibitor, to PTX for the treatment of metastatic TNBC. The administration of 90 mg/m^2^ (qw × 3 i.v.) along with 400 mg capivasertib (bid p.o.) every 28 days resulted in an improvement in median PFS from 4.2 to 5.9 months and median OS from 12.6 to 19.1 months compared to PTX plus placebo. In another phase II study, Gonzalez-Angulo et al.^23^ investigated the effect of the combination of everolimus, the orally administered analog of the mTOR inhibitor rapamycin (RAP), with PTX in a neoadjuvant sequential regimen containing anthracyclines on TNBC. Patients with primary TNBC were treated with T-FEC (PTX 80 mg/m^2^ qw × 12 i.v., followed by 5-fluorouracil 500 mg/m^2^, epirubicin 100 mg/m^2^, and cyclophosphamide 500 mg/m^2^ q3w × 4 i.v.) versus TR-FEC (PTX 80 mg/m^2^ qw × 12 i.v. and everolimus 30 mg qw × 12 p.o., followed by FEC). According to their results, the addition of everolimus was well tolerated and increased the 12-week clinical response rate from 29.6% to 47.8% and pathological complete response from 25.9% to 30.4%. Despite the enhancement in the clinical response rate, the pathological complete response rate did not show any significant improvement in this study.

The cumulative evidence suggests that despite the proven advantages of coadministration of an mTOR inhibitor such as RAP with PTX in the treatment of TNBC cell lines in vitro, the potential curative power of such a combination regimen remains untapped in vivo.^24^ Several factors may contribute to the failure to exploit the full potential of the combination of PTX and an mTOR inhibitor such as RAP. First, the oral administration of mTOR inhibitors is associated with complications such as poor absorption, poor permeability, and a potential first-pass effect.^25^ Additionally, the intravenous administration of PTX in the form of Taxol requires the use of Cremophor EL and ethanol for solubilization.^26^ This not only triggers life-threatening hypersensitivity reactions but also contributes to dose-limiting toxicities (DLT), despite premedication.^27^ Furthermore, the DLTs caused by the addition of mTOR inhibitors to certain treatment regimens may constrain efficacy and hinder dose escalation.^28,29^ Lastly, the disparity in the pharmacokinetics may result in tumor exposure to varying ratios of the combined drugs, which, in specific instances, might exhibit an antagonistic rather than synergistic effect. Campone et al.^28^ assessed the pharmacokinetic profiles of everolimus (15 mg p.o.) and PTX (80 mg/m^2^ i.v.) either in combination or alone in patients with advanced malignancies. They observed no substantial changes in *t*_max_, *C*_max_, and AUC_last_ of PTX and everolimus when administered in combination compared to those when administered alone. However, the two drugs exhibited significantly different terminal half-lives (*t*_1/2, PTX_ ≈ 7.6–8.3 h, *t*_1/2, EVL_ ≈ 24.6–29.5 h), indicating markedly distinct elimination rates, which consequently lead to exposure to variable combination ratios.

The concept of ratiometric dosing, initiated by Mayer and Janoff at Celator Pharmaceuticals, suggests the application of nanotechnology to deliver a synergistic ratio of a drug combination as an alternative to the administration of conventional free drug cocktail.^30–35^ Using this strategy, the pharmacokinetic profiles of the drugs in combination are synchronized by coencapsulation, and a synergistic ratio can be maintained in plasma.^33^ However, the challenge of encapsulating drugs with markedly different physicochemical properties, such as solubility and steric configuration, into the same nanocarrier using conventional encapsulation techniques, coupled with concerns regarding nanoparticle stability and varying release rates of the two drugs, poses significant obstacles to effectively exploiting this strategy.^36–44^

In recent years, our lab has developed oligo­(lactic acid)_*n*_ prodrugs of anticancer drugs to improve compatibility with the poly­(lactic acid) core of poly­(ethylene glycol)-*b*-poly­(lactic acid) (PEG-*b*-PLA) micelles.^45^ This strategy has led to enhancements in loading capacity, encapsulation efficiency and colloidal stability, extended drug release, and improved antitumor efficacy compared to micelles loaded with the parent anticancer drugs.^45–48^ Previous studies utilizing this platform have demonstrated that prodrug release and conversion can be fine-tuned based on the length of the oligo­(lactic acid) promoiety and the drug loading within the micelle.^45,46,49^ To date, this platform has primarily been applied for monotherapy. However, the attachment of an extended oligo­(lactic acid) chain to the parent drugs renders the resulting prodrugs more chemically and physically similar to one another than to the parent compounds. Consequently, coencapsulation of these prodrugs within the same nanocarrier is anticipated to enable harmonized coloading, coordinated corelease, synchronized pharmacokinetics, and maintenance of the desired combination ratio—addressing key challenges in ratiometric drug delivery. This concept—ratiometric control facilitated by prodrug assimilation—has not been previously explored in the literature, either by our group or others.

Here, we propose the application of Rapaxane for precise ratiometric dosing of PTX and RAP in combination therapy for the treatment of TNBC. Rapaxane is formulated by coloading a synergistic ratio of oligo­(lactic acid)_8_-conjugated prodrugs of paclitaxel (oLA_8_-PTX) and rapamycin (oLA_8_-RAP) into PEG_4 kDa_-*b*-PLA_2.2 kDa_ micelles. We hypothesize that the conjugation of the oligo­(lactic acid) promoiety to the parent drugs can assimilate their physicochemical properties, allowing for coencapsulation at the desired ratio, synchronizing drug release from the micelles, and exposing tumor cells to a synergistic drug ratio to enhance antitumor efficacy (Figure 1). Rapaxane is prepared using the PEG-assist method,^50^ a GMP-compliant, solvent-free fabrication approach developed in our laboratory. This work demonstrates, for the first time, that this method can be successfully applied to prepare combination prodrug-loaded micelles. We characterized Rapaxane in terms of its physicochemical properties and drug release profile. Subsequently, we compared its toxicity and antitumor efficacy in subcutaneous and orthotopic TNBC mouse models with the benchmark formulation Abraxane, as well as a combination of the parent drugs PTX and RAP, and monotherapies using the prodrugs.

**Figure 1:**
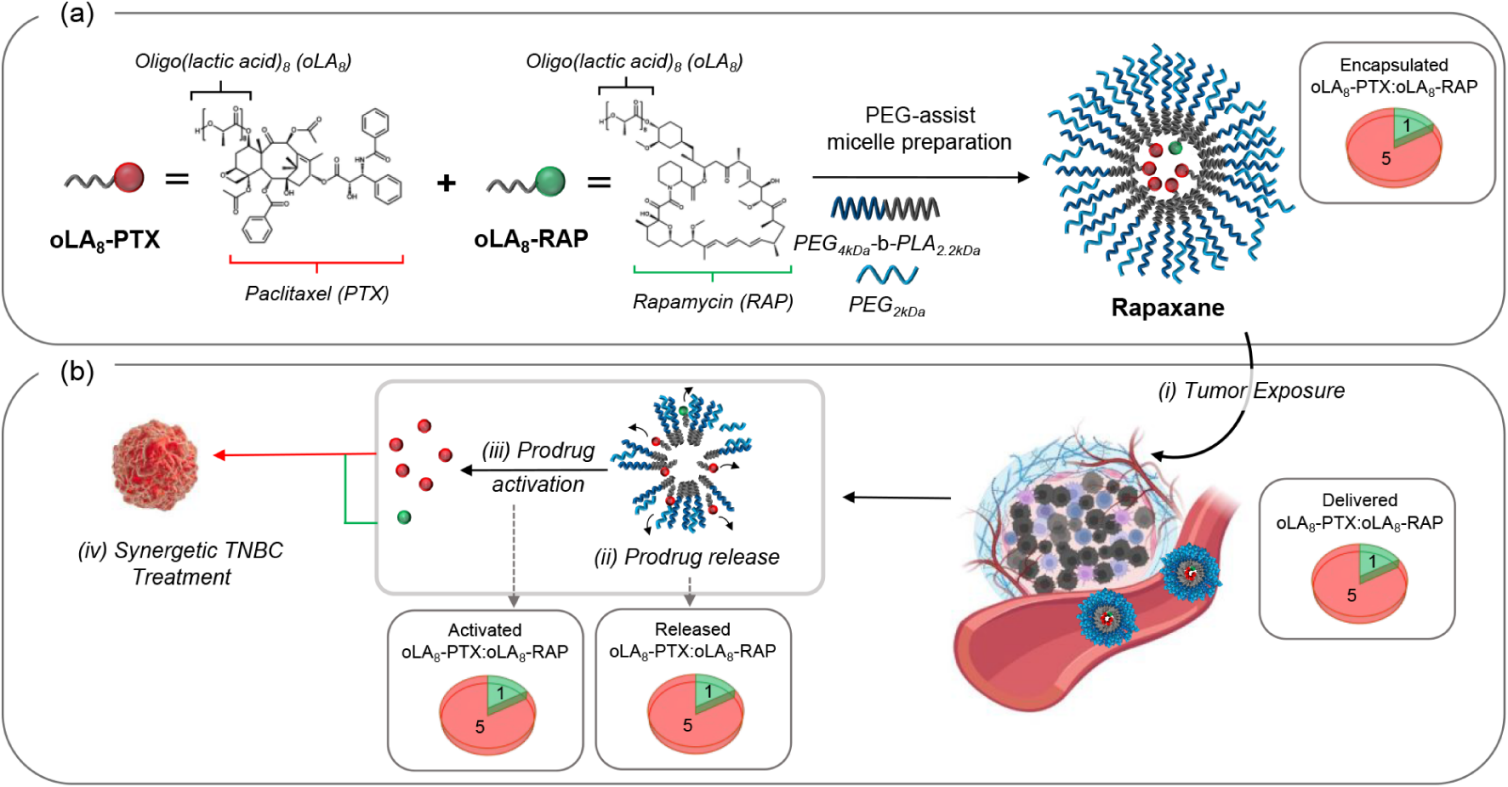
Schematic diagram of the ratiometric delivery system of two oligo­(lactic acid)_8_ anticancer prodrugs (oLA_8_-PTX and oLA_8_-RAP) for the treatment of triple-negative breast cancer (TNBC). (a) Preparation of Rapaxane, a PEG_4 kDa_-*b*-PLA_2.2 kDa_ polymeric micelle coloaded with oLA_8_-PTX and oLA_8_-RAP at the optimal synergistic ratio (5:1), as determined by combination index (CI) values in vitro against 4T1 and MDA-MB-231 cells. (b) Following intravenous (i.v.) injection, Rapaxane circulates in the bloodstream, exposing the tumor to the drugs at the intended molar ratio through synchronized release and activation.

Overall, this study encompasses the development of Rapaxane from GMP-compliant manufacturing to the preclinical evaluation of toxicity and efficacy, providing a foundation for further development of this formulation toward potential clinical applications.

## Results and Discussion

### oLA_8_-PTX and oLA_8_-RAP Show Synergy at the Same Combination Ratio as Their Parent Drugs

We first investigated the synergistic effect of PTX and RAP in combination therapy against two TNBC cell lines: 4T1 murine and MDA-MB-231 human breast cancer cells. To determine the most effective ratio of PTX and RAP, we analyzed cytotoxicity and calculated half-maximal inhibitory concentration (IC50) values following treatment with varying PTX and RAP ratios (PTX/RAP= 5:1, 4:2, 3:3, 2:4, 1:5) at concentrations ranging from 0 to 100 μM (Figure S1 and Table S1). 4T1 and MDA-MB-231 cells were incubated for 72 h with the PTX/RAP combinations, and cell viability was assessed using the CellTiter-Blue assay (Figure S1a,b).^46^

In both cell lines, PTX exhibited dose-dependent cytotoxicity with low IC50 values of 483 ± 26 nM in 4T1 cells and 1424 ± 74 nM in MDA-MB-231 cells, consistent with previously reported values in the literature.^51,52^ In contrast, RAP displayed minimal cytotoxicity within the tested concentration range, with IC50 values of 18147 ± 1030 nM in 4T1 cells and 137946 ± 1446 nM in MDA-MB-231 cells (Figure S1c,d). However, when PTX and RAP were combined, the IC50 values decreased for specific tested ratios compared to those of either drug administered alone. The 5:1 PTX:RAP ratio was the most potent, showing an IC50 of 139 ± 20 nM in 4T1 cells, while other ratios had IC50 values ranging from 217 to 867 nM (Figure S1a,c). Similar synergistic trends were observed in MDA-MB-231 cells, where the 5:1 PTX:RAP ratio was again the most effective, with the lowest IC50 value of 607 ± 24 nM (Figure S1b,d).

The data also suggest that increasing the RAP content in the combination beyond a certain ratio reduces its effectiveness compared to single PTX treatment in both cell lines. Combination index (CI) values, which classify interactions as antagonistic (CI > 1), additive (CI = 1), or synergistic (CI < 1), were calculated at different fractions of affected cells using CompuSyn software, based on the median-effect Chou and Talalay equation.^53^ In 4T1 cells, a synergistic effect of PTX and RAP was observed for most ratios except for 1:5, where the combination was less effective (Table S2, left column). At *F*_a_ = 0.5, 0.75, and 0.9, CI values were <1 for the 5:1, 4:2, 3:3, and 2:4 PTX:RAP ratios, indicating synergism between the two drugs.^53^ In MDA-MB-231 cells, only the 5:1 PTX:RAP ratio showed synergism, with CI values of 0.356 at *F*_a_ = 0.5, 0.275 at *F*_a_ = 0.75, and 0.212 at *F*_a_ = 0.9 (Table S2, right column).

Synergistic effects of combining rapamycin with paclitaxel against various cancer cell lines have also been demonstrated by other researchers. For instance, Aissat et al.^54^ reported a synergistic interaction between rapamycin and paclitaxel in SQ20B and HEP2 cells. They found that the synergistic effect was more pronounced when cells were first treated with paclitaxel, followed by rapamycin. The authors attributed these observations to the potential influence of rapamycin on the cell cycle progression and distribution. In another study, Shafer et al.^55^ demonstrated that simultaneous exposure of endometrial cancer cell lines, Ishikawa and ECC-1, to various doses of paclitaxel in combination with rapamycin resulted in a significant synergistic antiproliferative effect. Their findings indicated that rapamycin alone did not induce apoptosis; however, combined treatment with paclitaxel enhanced apoptosis compared with paclitaxel alone. The cotreatment with rapamycin and paclitaxel also led to a reduction in the phosphorylation of S6 and 4E-BP1, two critical downstream targets of the mTOR pathway. Additionally, rapamycin was shown to downregulate hTERT mRNA expression, while paclitaxel alone had no effect on telomerase activity. Paclitaxel was found to increase tubulin polymerization and acetylation, with rapamycin further enhancing this effect.

Next, we performed a viability assay on 4T1 and MDA-MB-231 cell lines following treatment with oligo­(lactic acid)_8_–paclitaxel (oLA_8_-PTX) and oligo­(lactic acid)_8_–rapamycin (oLA_8_-RAP) to confirm whether the combination of these prodrugs shows a trend similar to that of the PTX and RAP combination (Figure 2 and Table S3). Both oLA_8_-PTX and oLA_8_-RAP were synthesized according to previously published protocols.^45–47^ Briefly, oLA_8_-PTX was prepared via esterification at the 7-OH position of PTX with oligo­(lactic acid)_8_ using dicyclohexylcarbodiimide coupling, while oLA_8_-RAP was synthesized by conjugating oligo­(lactic acid)_8_ to the C-40 hydroxyl position of RAP. The chemical structures of oLA_8_-PTX and oLA_8_-RAP were confirmed by ^1^H NMR and mass spectroscopy (LC-MS) (Figures S2–S5).

**Figure 2:**
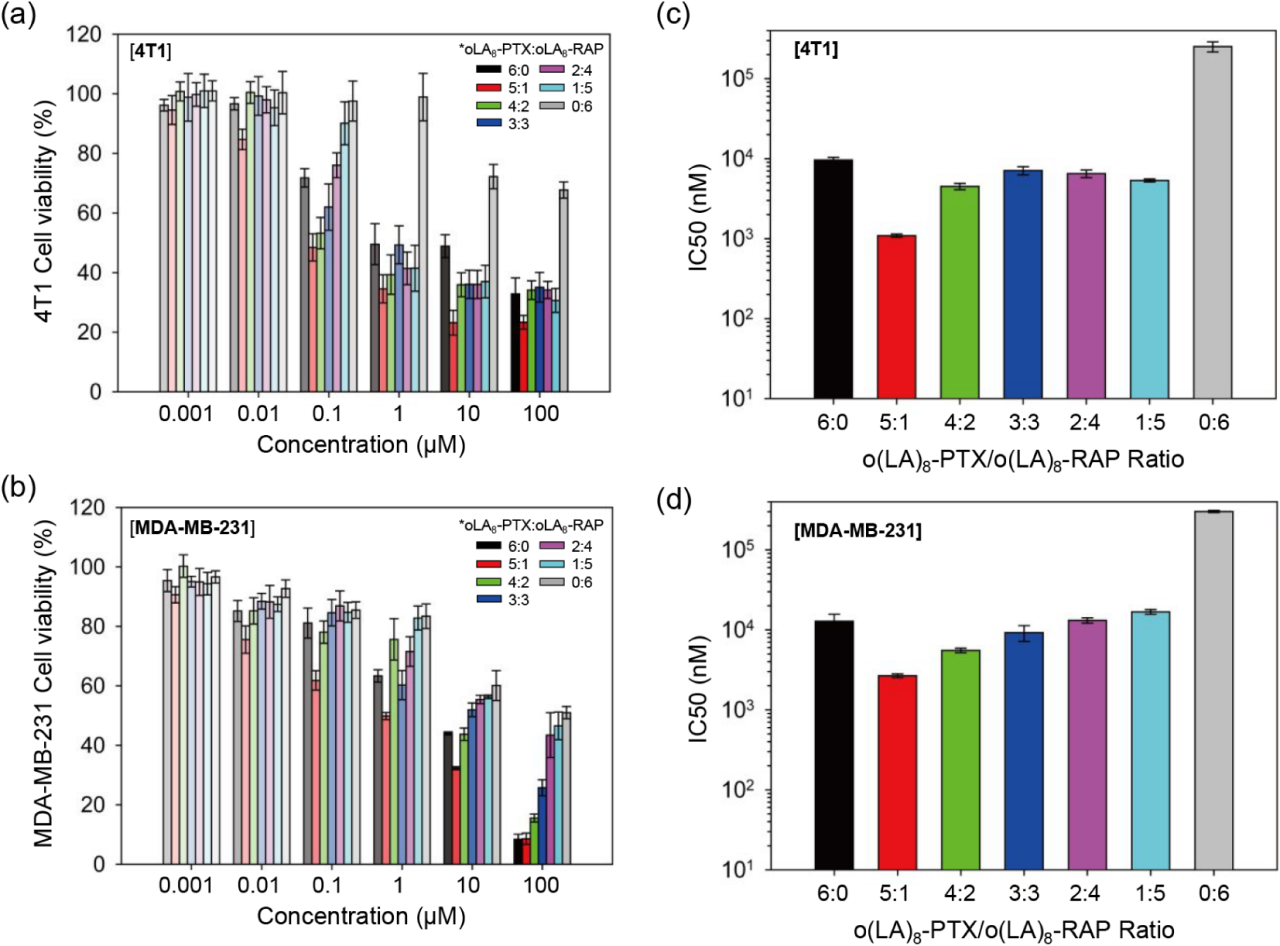
Synergistic cytotoxic effect of oLA_8_-PTX and oLA_8_-RAP at various ratios (6:0 to 0:6) against 4T1 (murine breast cancer) and MDA-MB-231 (human breast cancer) cell lines. Cell viability of (a) 4T1 cells and (b) MDA-MB-231 cells treated with different concentrations (0–100 μM) and ratios of oLA_8_-PTX and oLA_8_-RAP, incubated at 37 °C for 72 h. Shading corresponds to the concentration. Cytotoxicity was assessed using the CellTiter Blue Viability Assay. IC50 values for the different oLA_8_-PTX:oLA_8_-RAP ratios in (c) 4T1 cells and (d) MDA-MB-231 cells (*n* = 8 replicates; mean ± S.D.), derived from the cytotoxicity data shown in panels (a) and (b).

We treated the cells with various oLA_8_-PTX and oLA_8_-RAP combination ratios (5:1, 4:2, 3:3, 2:4, 1:5) at concentrations ranging from 0 to 100 μM to calculate cytotoxicity and IC50 values (Figure 2 and Table S3). In both cell lines, single oLA_8_-PTX treatments induced dose-dependent cytotoxicity but with higher IC50 values compared to PTX: 9628 ± 719 nM for 4T1 and 12 829 ± 2839 nM for MDA-MB-231. This increase is likely due to the oligo­(lactic acid) chain, which requires hydrolysis over time to release the active cytotoxic species.^45,46^ Single oLA_8_-RAP treatment showed negligible cytotoxic effects in both cell lines, with IC50 values of 252 021 ± 36 392 nM for 4T1 and 301 961 ± 8947 nM for MDA-MB-231.

Combination treatments of oLA_8_-PTX and oLA_8_-RAP demonstrated results similar to those of the PTX and RAP combinations. The 5:1 ratio exhibited the highest cytotoxicity against TNBC cells, with the lowest IC50 values of 1088 ± 53 nM for 4T1 and 2666 ± 144 nM for MDA-MB-231, compared to other ratios or single treatments. This suggests that conjugation with oLA_8_ does not impact the synergistic effect of the parent drugs, possibly due to the synchronized conversion of the two prodrugs to active species or parent drugs. The calculated combination index (CI) values of the oLA_8_-PTX and oLA_8_-RAP combinations supported the cytotoxicity results (Table S4), showing a synergistic effect with CI values of <1 at *F*_a_ = 0.5, 0.75, and 0.9 in both cell lines.

### oLA_8_-PTX and oLA_8_-RAP Can Be Coloaded, Released, and Converted Ratiometrically

Next, we prepared oLA_8_-PTX and oLA_8_-RAP-coloaded PEG-*b*-PLA polymeric micelles at different ratios using the recently developed fabrication method in our lab, named PEG-assist.^50^ This method involves simple steps of heating, mixing, and cooling. In essence, micelle preparation entails heating to dissolve PEG-*b*-PLA along with o­(LA)_*n*_-prodrugs in PEG of a specific molecular weight as the solvent, followed by hydration and cooling. Subsequent steps include sterile filtration and freeze-drying. The PEG-assist approach holds promise for large-scale production of drug-loaded polymeric micelles for clinical translation due to several advantages: (i) no requirement for toxic organic solvents during fabrication (excluding polymer synthesis); (ii) simplicity, rapidity, and cost-effectiveness of the procedure; (iii) high encapsulation efficiencies and uniform particle size distribution; (iv) controllability of micelle characteristics through temperature and composition adjustments; (v) potential utilization of PEG as a lyoprotectant during freeze-drying, obviating the need for its removal. Briefly, a 1:1:20 w/w/w ratio of prodrugs:PEG_4 kDa_-*b*-PLA_2.2 kDa_:PEG2000 (target prodrug loading: 50 wt %) was mixed and dissolved at 60 °C for 1 h, followed by the addition of prewarmed water (60 °C) and cooling to room temperature. The free drug was removed by centrifugation at 10000*g* for 5 min. Single prodrug-loaded micelles were also prepared using the same method. All micelle formulations exhibited uniform hydrodynamic sizes between 98 and 118 nm with a low polydispersity index (PDI < 0.25), indicating that coloading the prodrugs had no impact on micelle size or uniformity (Figure 3a and Table S5). These size and PDI values were maintained for 28 days in deionized water (Figure S6). These multiple-prodrug-loaded micellar formulations remained stable at room temperature for over 2 months, with no precipitation observed. We have previously demonstrated that oLA_8_-PTX and oLA_8_-RAP-loaded PEG-*b*-PLA polymeric micelles also preserve the chemical stability of the prodrugs.^56^ Additionally, micelle size was analyzed in phosphate-buffered saline (PBS, pH 7.4) and Dulbecco’s modified Eagle’s medium (DMEM). No significant aggregation or precipitation was observed, demonstrating the high colloidal stability of both single- and multiprodrug-loaded micelles in physiological media (Figure S7 and Table S6).

**Figure 3:**
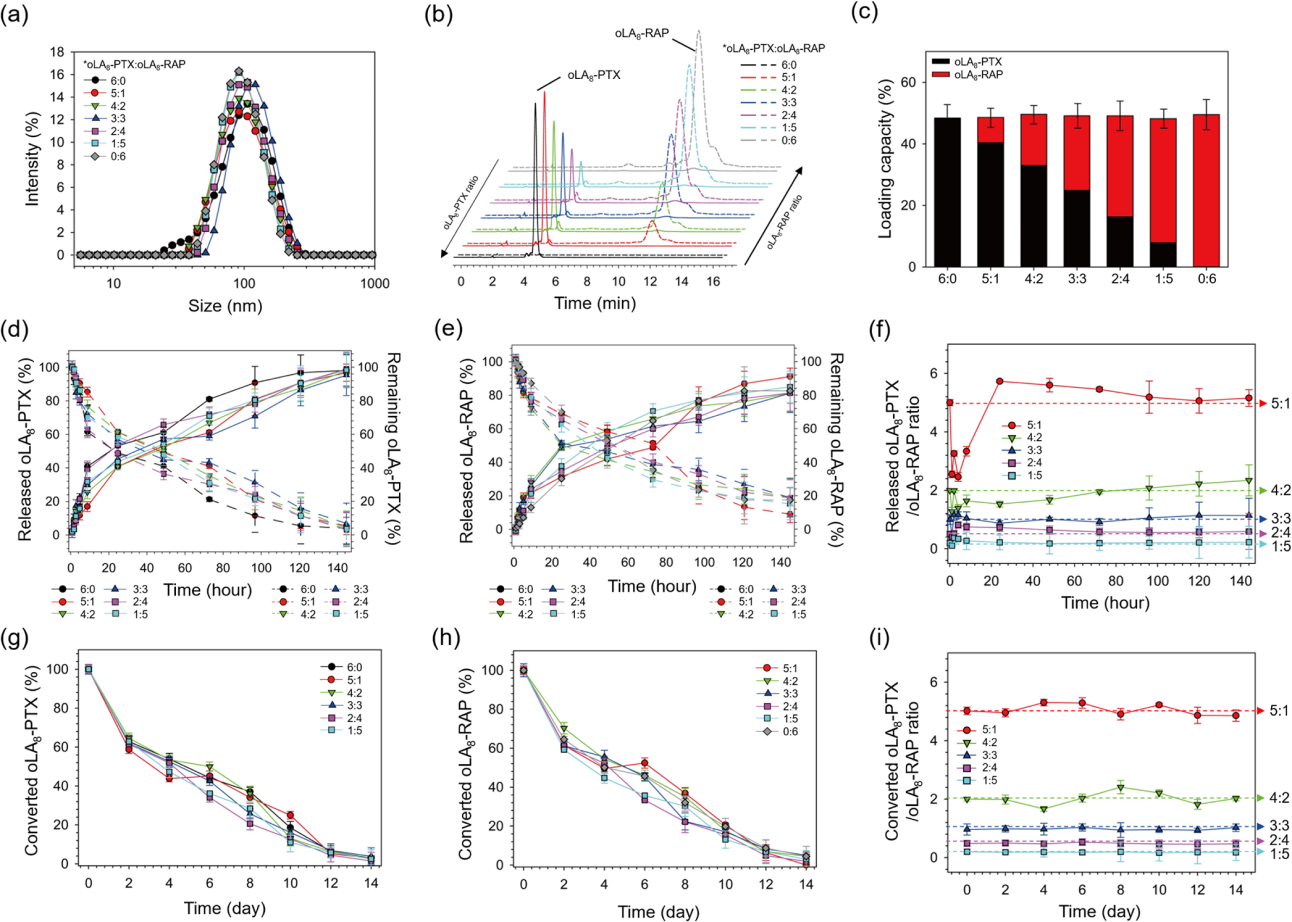
Characterization of PEG_4 kDa_-*b*-PLA_2.2 kDa_ polymeric micelles encapsulating various ratios of oLA_8_-PTX and oLA_8_-RAP prodrugs. (a) Average hydrodynamic size (intensity distribution) measured by dynamic light scattering (DLS). Mean values and standard deviations were calculated from triplicate measurements. (b) Reverse-phase HPLC chromatograms and (c) quantified amounts of encapsulated oLA_8_-PTX and oLA_8_-RAP within polymeric micelles (mean ± S.D., *n* = 3). In vitro release profiles of (d) oLA_8_-PTX and (e) oLA_8_-RAP from micelles loaded with various prodrug ratios in PBS (pH 7.4) at 37 °C over 144 h (mean ± S.D., *n* = 3). Left *y*-axis (solid line): Prodrug released from micelles. Right *y*-axis (dashed line): Prodrug remaining within micelles. (f) Ratio of released oLA_8_-PTX to oLA_8_-RAP in PBS (pH 7.4) at selected time points. In vitro conversion profiles of (g) oLA_8_-PTX and (h) oLA_8_-RAP released from polymeric micelles over 14 days in PBS (pH 7.4) at 37 °C (mean ± S.D., *n* = 3). (i) Ratio of converted oLA_8_-PTX to converted oLA_8_-RAP at specific time points after release in PBS (pH 7.4).

Reverse-phase HPLC (RP-HPLC) analysis revealed that the micelles retained the specific oLA_8_-PTX:oLA_8_-RAP ratios initially loaded, with encapsulation efficiencies of ∼99%, translating to over 48% weight loading for both prodrugs (Figure 3b,c and Table S5). This high loading and encapsulation can be attributed to the strong interaction between the PLA core and the oLA_8_ prodrugs.^46^ As a control, we prepared PTX and RAP-coloaded micelles using the thin-film hydration method, since RAP is insoluble in PEG2000. The parent drug-loaded micelles showed lower encapsulation efficiencies (∼70% w/w, corresponding to 35 wt % total drug loading) and instability, with both drugs precipitating from the solution within 24 h at room temperature (Table S7).

We also freeze-dried the samples and analyzed the reconstituted micelles. Following the preparation of drug-loaded micelles and the removal of free prodrug by centrifugation, the samples were frozen at −80 °C and subsequently freeze-dried using a shelf freeze-dryer (VirTis, SP Scientific) set at −35 °C and 150 μBar. All formulations maintained their size, PDI, and encapsulation efficiency, as well as the loaded drug ratio, matching the initial samples before freeze-drying (Table S8). These results confirm that oLA_8_ prodrug combinations can be successfully coloaded into PEG_4 kDa_-*b*-PLA_2.2 kDa_ micelles with desirable ratios, high stability in biological solutions, and preservation of physicochemical properties post-freeze-drying using the PEG-assist method.

We next investigated whether the synchronized release of the two prodrugs from the micelles could maintain the loaded ratio throughout the release. Our results showed that the release profiles of oLA_8_-PTX (Figure 3d) and oLA_8_-RAP (Figure 3e) were highly similar across all tested ratios, maintaining consistent drug ratios in the release media (Figure 3f). Both prodrugs require conversion to their active forms,^45,46^ so we monitored their conversion rates by measuring the disappearance of the prodrugs from the media over time. The conversion rates of oLA_8_-PTX (Figure 3g) and oLA_8_-RAP (Figure 3h) were synchronized, ensuring that the converted drug ratio (Figure 3i) matched the initially loaded ratio.

In summary, our data demonstrate that the oLA_8_ prodrug platform enables coloading at precise ratios, synchronized release, and consistent conversion, making it highly suitable for ratiometric delivery of PTX and RAP in combination therapies.

Ratiometric dosing, introduced by Mayer et al., demonstrated a positive correlation between the prevalence of synergy and antagonism in vitro and the degree of antitumor activity at the maximum tolerated dose (MTD) in vivo using liposomal cytarabine/daunorubicin in various leukemia models.^31^ In ratiometric dosing, the typical approach involves identifying an optimal drug ratio using combination analysis models, such as the Chou–Talalay model,^57,58^ followed by loading the drugs into nanocarriers that can maintain the ratio in plasma.^33^ Coencapsulation of the combination is expected to synchronize the pharmacokinetics (PK) of the drugs, thereby ensuring that the tumor is exposed to the desired synergistic ratio, which may lead to reduced dose-limiting toxicities (DLTs), enable dose escalation, and enhance the antitumor response.

However, several challenges and concerns are associated with this strategy:

(i) Exact ratiometric loading cannot be assured using conventional encapsulation techniques.^44^ Loading drugs with drastically different physicochemical properties, such as solubility and steric configuration, into the same nanocarrier presents significant challenges.^36–43^ One suggested solution is to incorporate the components into separate hydrophobic cores with similar surface properties for successful ratiometric encapsulation.^40^ While this approach enables precise ratiometric loading, the achieved loading capacity is highly limited.^40^ On the other hand, increasing the drug loading may reduce the encapsulation efficiency, which can affect the ratiometric coencapsulation.^59^ Therefore, to achieve the desired ratio, the encapsulation efficiencies of the components must be highly comparable.^59^
(ii) Disparities in the release profiles of coencapsulated drugs can lead to significant deviations from the desired synergistic ratio. It has been suggested that, for certain carrier/drug combinations, the components retain their individual release profiles even after ratiometric encapsulation into the same nanoparticle.^59^ To address potential issues arising from unsynchronized release and its impact on the plasma ratio, one may propose the use of a highly stable nanoparticulate delivery system, assuming adequate retention of both drugs within the nanocarrier and extended circulation times for both drugs.^60^ This strategy can be effective when the delivery system relies on the enhanced permeability and retention (EPR) effect; however, two complications arise. First, stable nanoparticles may fail to efficiently release their cargo at the tumor site despite high accumulation via the EPR effect.^60^ Second, many nanoparticulate systems exhibit burst release and instability in the bloodstream. Even for highly stable particles, approximately 50% of the encapsulated drug is released within the first 24 h in vitro.^60^
(iii) Disparities in the PK characteristics of the released drugs can further lead to significant deviations from the desired synergistic ratio. Even if the drugs are released in the intended ratio, the pharmacokinetic profiles of the free drugs will eventually influence the overall ratio. This is particularly important when the two drugs in their free form have significantly different PK profiles, such as paclitaxel and everolimus (EVL), which exhibit markedly distinct terminal half-lives (*t*_1/2, PTX_ ≈ 7.6–8.3 h, *t*_1/2, EVL_ ≈ 24.6–29.5 h), indicating differing elimination rates and resulting in variable exposure to the combination ratio.^28^

In this context, utilizing a platform like the one introduced here, which addresses the aforementioned challenges by assimilating the physicochemical properties of the combination components, ensuring precise coencapsulation, harmonizing the release profiles from the nanoparticles, and synchronizing the conversion to the parent drugs, appears to be an effective approach.

### Cytotoxicity of oLA_8_-PTX/oLA_8_-RAP Coloaded Micelle against TNBC Cell Lines Is Ratio Dependent

We further evaluated the cellular toxicity of micellar formulations against the 4T1 and MDA-MB-231 cell lines (Figure 4). Cells were treated with single- and multiprodrug-loaded micelles (0–100 μM) for 72 h at 37 °C. PBS-treated cells served as the negative control.

**Figure 4:**
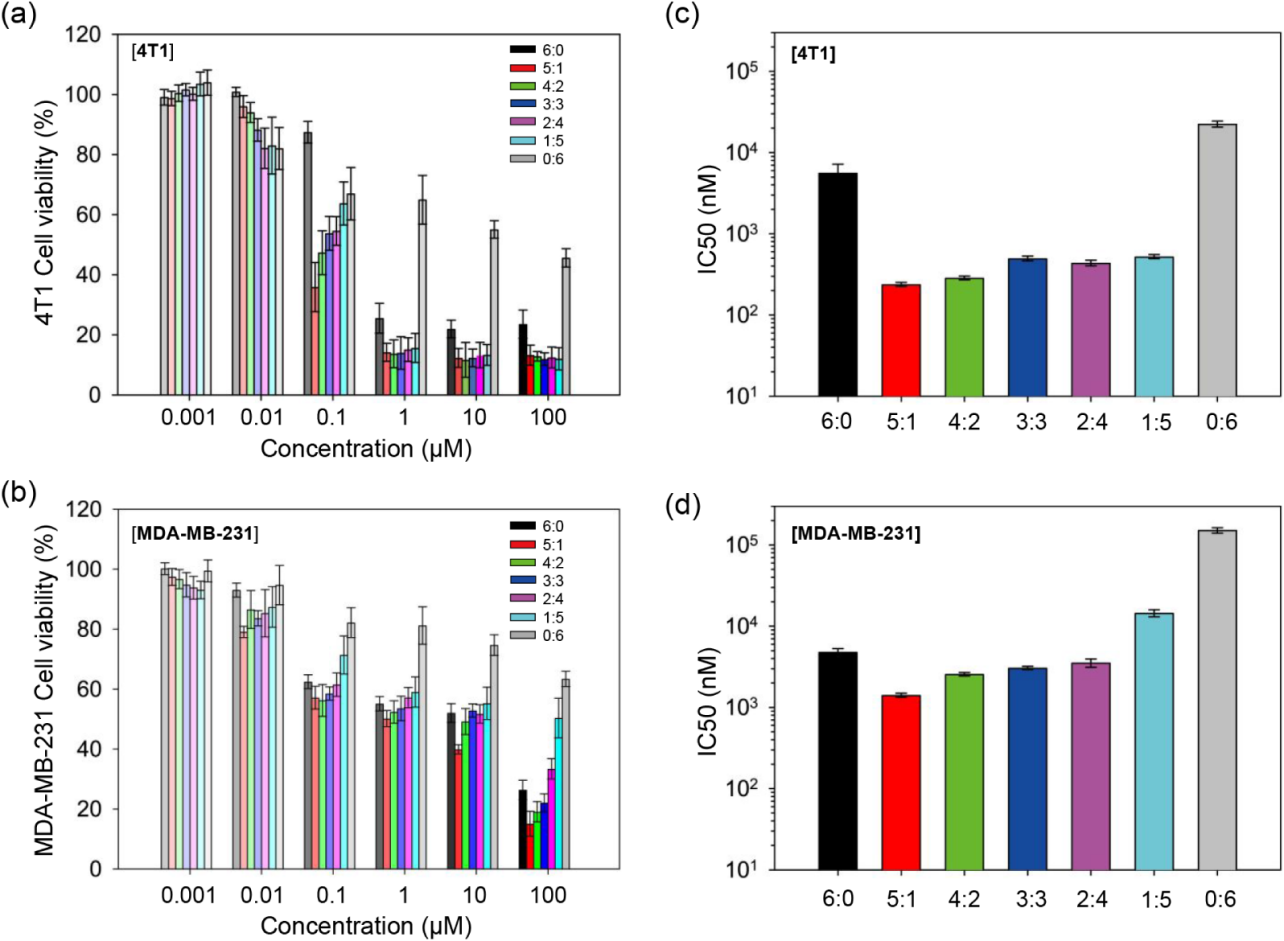
Cytotoxicity analysis of PEG_4 kDa_-*b*-PLA_2.2 kDa_ polymeric micelles coloaded with various ratios of oLA_8_-PTX and oLA_8_-RAP against 4T1 and MDA-MB-231 cells. Cell viability of (a) 4T1 and (b) MDA-MB-231 cells after treatment with micelles coloaded with different ratios of oLA_8_-PTX and oLA_8_-RAP incubated for 72 h at 37 °C. The concentrations were determined based on the encapsulated oLA_8_-PTX and oLA_8_-RAP. IC50 values for micelles loaded with various oLA_8_-PTX:oLA_8_-RAP ratios against (c) 4T1 and (d) MDA-MB-231 cells (*n* = 8 replicates; mean ± S.D.), derived from cell viability results shown in panels (a) and (b).

All micellar formulations demonstrated significant dose-dependent toxicity, with the highest toxicity observed in cells treated with the 5:1 ratio of oLA_8_-PTX/oLA_8_-RAP coloaded micelles for both 4T1 and MDA-MB-231 cells (Figure 4a,b). Single oLA_8_-PTX-loaded micelles exhibited IC50 values of 5607 ± 1597 nM for 4T1 and 4813 ± 504 nM for MDA-MB-231 cells. These values are higher than the IC50 values for the clinical benchmark formulation of PTX, Abraxane, as reported by Tanei et al.^51^ and Chen et al.^52^ The higher IC50 may be attributed to the high stability of oLA_8_-PTX-loaded micelles, as well as the necessity of conversion to the active parent drug or metabolites for cytotoxicity. Single oLA_8_-RAP-loaded micelles demonstrated negligible cytotoxicity, with significantly higher IC50 values (22 479 ± 1884 nM for 4T1 and 151 525 ± 11 631 nM for MDA-MB-231) (Figure 4c,d and Table S9).

Overall, coloaded oLA_8_-PTX/oLA_8_-RAP micelles demonstrated enhanced cytotoxic efficacy against 4T1 cells compared to single oLA_8_-PTX or oLA_8_-RAP across all tested ratios (Figure 4c). In MDA-MB-231 cells, all formulations, except for the 1:5 coloaded ratio micelles, exhibited lower IC50 values than single oLA_8_-PTX or oLA_8_-RAP (Figure 4d). The IC50 values for all samples against both cell lines are summarized in Table S9.

These results suggest that the synergistic ratio of free drugs and prodrugs, particularly at a 5:1 ratio, translates into superior performance in polymeric micelles loaded with multiple prodrugs at the same ratio in vitro, utilizing our oLA_8_-prodrug coloading strategy. In this context, the 5:1 oLA_8_-PTX/oLA_8_-RAP coloaded micelles exhibit significant potential for treating TNBC. This formulation preserves the synergistic effects of PTX and RAP by maintaining the initial specific ratios of coloaded prodrugs throughout encapsulation, release, and activation. However, it is important to note that this may not apply to all ratios, drug combinations, and nanocarriers. In a recent study, Detappe et al.^61^ introduced a bottlebrush prodrug (BPD) platform designed to coload mixtures of bortezomib, pomalidomide, and dexamethasone. This platform exhibited in vitro synergistic, additive, or antagonistic patterns, which were distinct from those of the corresponding free drugs in multiple myeloma models. They observed that free drugs and BPDs had different combination indexes, suggesting that directly translating free-drug ratios to nanocarriers could be detrimental in their system. Consequently, they proposed new design principles for combination nanomedicines based on the synergistic activity of the nanomedicine rather than free drugs. The key advantage of our proposed platform is its ability to ensure accurate coloading and synchronized release and conversion, allowing the synergistic pattern of the coloaded nanomedicine to mirror that of the free drugs. This makes the synergistic free-drug ratios translatable to nanocarriers, as supported by the presented data. We expect that this synergistic ratio will also translate to superior in vivo performance of our formulation compared to both synergistic free-drug ratios and single prodrug-loaded polymeric micelles, as will be discussed in the upcoming sections.

### Rapaxane Demonstrates Superior Efficacy in a Subcutaneous 4T1 Breast Cancer Mouse Model Compared to Abraxane and Single Prodrug-Loaded Formulations

Considering the promising results of cellular experiments, we selected the 5:1 coloaded ratio of oLA_8_-PTX/oLA_8_-RAP (designated as **Rapaxane**) for further evaluation and demonstrated its therapeutic efficacy in a subcutaneous 4T1 breast cancer mouse model. Prior to conducting in vivo experiments, we assessed the hemolytic properties of Rapaxane to confirm its safety for intravenous administration. Red blood cells collected from mice were incubated for 1 h at 37 °C with Rapaxane (0.1 mg/mL), other micellar formulations, and Abraxane. Saline and Triton X-100 served as negative and positive controls, respectively. While parent PTX/RAP coloaded micelles exhibited slightly increased hemolytic activity compared to saline, Rapaxane demonstrated negligible hemolytic activity, indicating excellent safety for intravenous injection (Figure S8).

We then evaluated the therapeutic efficacy of Rapaxane against TNBC in a subcutaneous 4T1 breast cancer mouse model. On day 14, 4T1 cells were injected subcutaneously into the flanks of mice to induce tumors. After 2 weeks, when tumor volumes reached approximately 100 mm^3^, the tumor-bearing mice were treated with saline, oLA_8_-PTX (single oLA_8_-PTX-loaded micelles), oLA_8_-RAP (single oLA_8_-RAP-loaded micelles), PTX/RAP (5:1 ratio of PTX:RAP coloaded micelles), Abraxane, and Rapaxane at doses of 50 mg/kg of PTX or oLA_8_-PTX and 10 mg/kg of RAP or oLA_8_-RAP. Treatments were administered via tail vein injections on days 0, 7, and 14 (Figure 5a).

**Figure 5:**
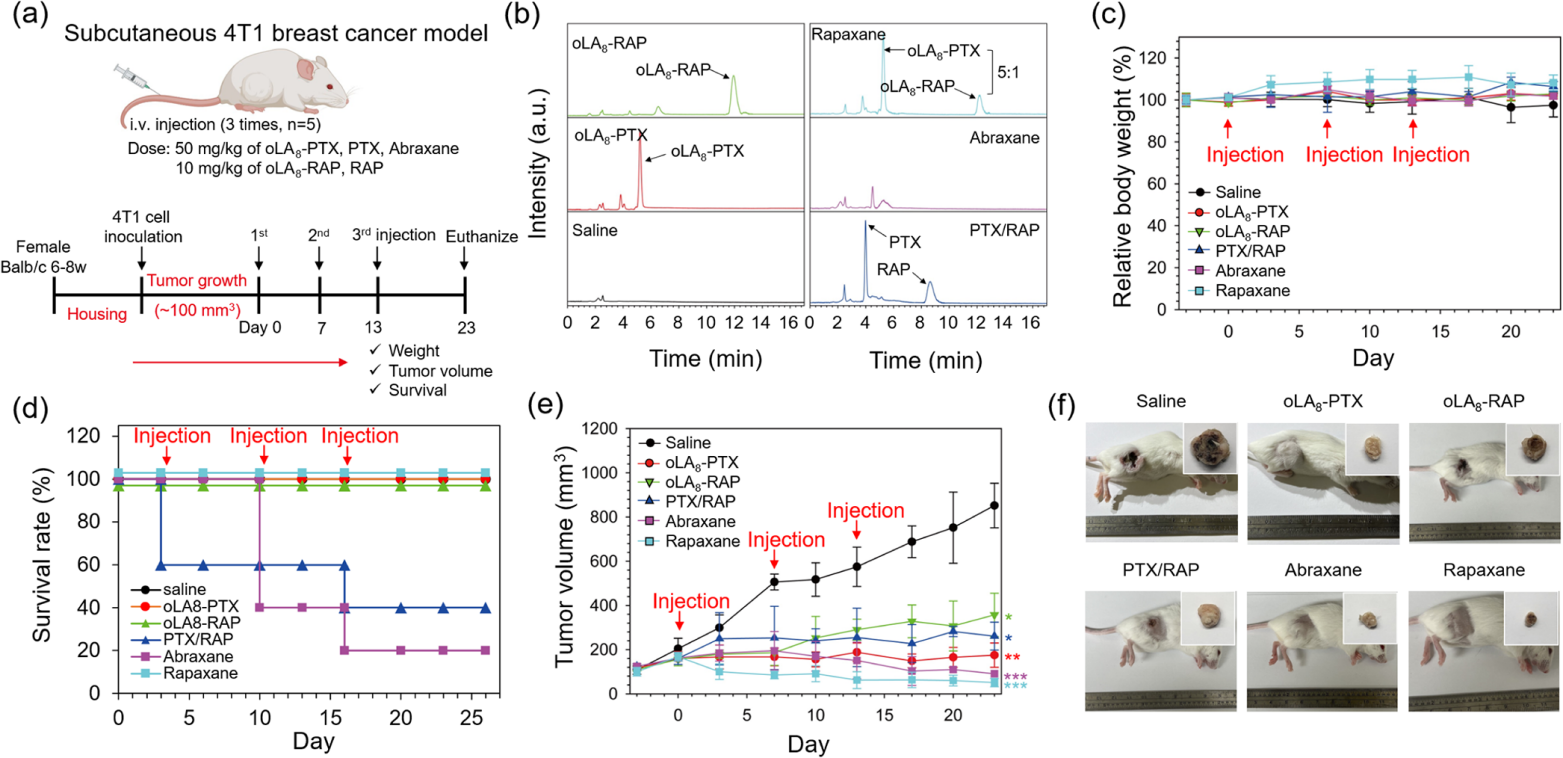
Therapeutic efficacy of Rapaxane in a subcutaneous 4T1 breast cancer mouse model. (a) Schematic representation of the development of the subcutaneous 4T1 breast cancer mouse model and details of the efficacy experimental design, including the dosing schedule. (b) Representative RP-HPLC chromatograms of mouse plasma 5 min after first i.v. injection of saline, oLA_8_-PTX, oLA_8_-RAP, PTX/RAP, Abraxane, and Rapaxane. 50 mg/kg of PTX equivalent and 10 mg/kg of RAP equivalent doses were employed for all the treatments. (c) Relative changes in body weight, (d) survival rate, and (e) subcutaneous tumor volume changes following treatment with saline, oLA_8_-PTX, oLA_8_-RAP, PTX/RAP, Abraxane, and Rapaxane (*n* = 5, *p ≤ 0.05, **p ≤ 0.01, ***p ≤ 0.001). (f) Representative photographs of mice and harvested tumors (inset) on Day 23.

We verified the prodrug ratio in mouse plasma to ensure that Rapaxane retained its initially loaded prodrug ratio following administration. After the first tail vein injection of Rapaxane and other formulations into mice bearing subcutaneous 4T1 breast cancer tumors, blood samples were collected via the retro-orbital vein 5 min post injection and analyzed using RP-HPLC. The results demonstrated that intravenously administered Rapaxane preserved its original coloaded prodrug ratio in plasma, with no indication of rapid alterations in the ratio after injection (Figure 5b).

We monitored changes in body weight, survival rates, tumor volume, and any observable toxic effects (Figure 5c–e). The body weights of mice treated with saline, oLA_8_-PTX, oLA_8_-RAP, and Abraxane remained unchanged throughout the study. In contrast, mice treated with PTX/RAP and Rapaxane exhibited a slight increase in their body weight for unknown reasons (Figure 5c). Notably, Rapaxane-treated mice demonstrated a significantly higher survival rate (100%) over 23 days compared to PTX/RAP (40%) and Abraxane (20%) treated groups. Mice treated with micellar oLA_8_-PTX and oLA_8_-RAP also showed 100% survival, suggesting that oligo­(lactic acid)_8_ conjugation mitigates the acute toxicity of the parent drugs (Figure 5d).

Rapaxane significantly reduced tumor volumes compared to other treatments (Figure 5e). Tumor growth was inhibited following the first injection, and the reduction in the tumor size was maintained throughout the experiment. At the study’s conclusion, tumor volumes in the Rapaxane-treated group averaged 51.0 ± 22.7 mm^3^, dramatically smaller than those in saline (852.0 ± 101 mm^3^), oLA_8_-PTX (175 ± 55 mm^3^), oLA_8_-RAP (357 ± 98 mm^3^), PTX/RAP (261 ± 63 mm^3^), and Abraxane (91 mm^3^) treated groups (Figure 5f). These findings highlight the superior antitumor efficacy of Rapaxane compared to other formulations. Considering the incomplete survival observed in the PTX/RAP and Abraxane treatment groups, it should be noted that tumor volumes for these groups were calculated based on the average tumor size of the surviving animals at each time point, and all statistical analyses were performed accordingly.

The synergistic effects of rapamycin and paclitaxel have been extensively investigated both preclinically and clinically across various cancers.^28,62–65^ The activation of Akt following paclitaxel treatment, which is believed to contribute to drug resistance,^66,67^ along with the role of mTOR as a critical cell survival protein,^62^ has been a central focus in understanding the mechanism underlying the synergy between rapamycin and paclitaxel. Blanco et al.^68^ investigated the codelivery of rapamycin and paclitaxel to tumors via nanoparticles and proposed that this combination synergistically targets the PI3K/Akt/mTOR pathway by inhibiting the Akt phosphorylation feedback loop. Their findings demonstrated that codelivery of rapamycin and paclitaxel resulted in reduced phosphorylation of S6, S6K, and 4E-BP1, while also leading to a decrease in pAkt levels. Furthermore, the combination therapy inhibited the phosphorylation of PRAS40, Bad, and GSK3β—proteins whose activation is typically increased by rapamycin treatment alone. While the detailed mechanism of Rapaxane’s action warrants further investigation, we believe it is likely similar to the mechanism proposed by Blanco et al.,^68^ as the prodrugs in our formulation must be converted to their parent drugs and active metabolites for therapeutic action.

### Toxicological Studies Suggest a Higher Tolerated Dose for Rapaxane Compared to Abraxane

Following the observation of acute toxicity associated with Abraxane in the subcutaneous model, we conducted a dose titration toxicity study to determine the maximum tolerated dose (MTD) for both Rapaxane and Abraxane. Healthy female and male mice were used in this study, starting with an initial dose of 15 mg/kg PTX-equivalent, which was increased by 15 mg/kg weekly (Figure 6a). At a dose of 30 mg/kg, both Rapaxane and Abraxane were well tolerated. However, upon escalating the dose to 45 mg/kg, 50% mortality was observed in the Abraxane group (males and females combined, Figure 6b), establishing 45 mg/kg as the LD50 for Abraxane, which was consistent with findings from the subcutaneous model. In contrast, Rapaxane was well tolerated at 45 mg/kg, prompting further dose escalation for this formulation while the Abraxane dose was reduced back to 30 mg/kg in week 4 and maintained in the following weeks. The study was conducted over 8 weeks, consisting of 4 weeks of treatment and 2 weeks of rest, followed by 2 additional weeks of treatment. Body weight changes were not significant throughout the study in either the female or male groups (Figure 6c). Our findings indicated that Rapaxane was well tolerated up to 90 mg/kg, while the maximum tolerated dose for Abraxane was 30 mg/kg per week without acute toxicity.

**Figure 6:**
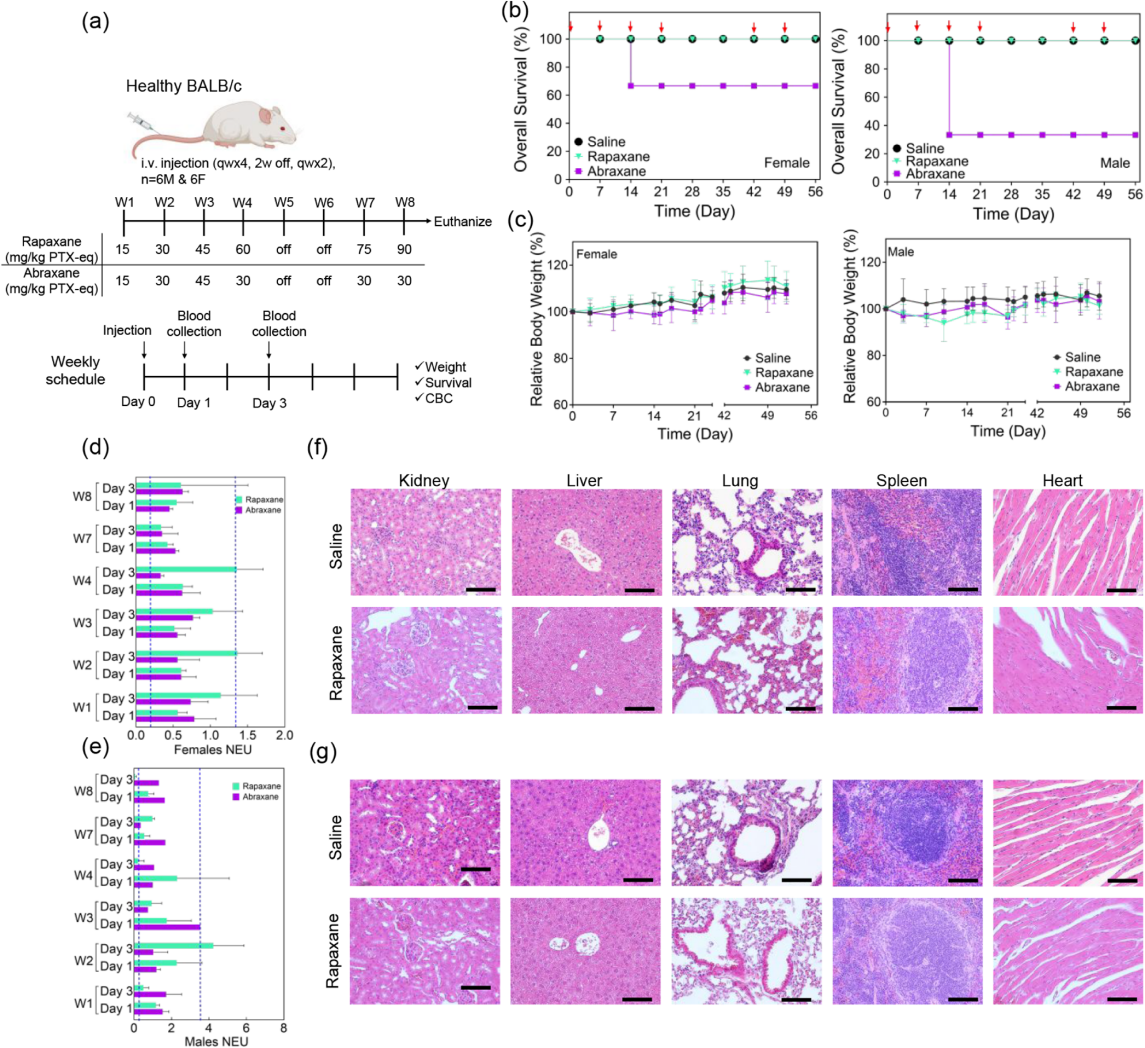
Toxicity study of Rapaxane and Abraxane in healthy female and male BALB/c mice. (a) Dose titration of Rapaxane and Abraxane was initiated at 15 mg/kg PTX equivalent, with increases of 15 mg/kg weekly over 4 weeks of administration, followed by 2 weeks off and then 2 additional weeks of administration. The administered doses for Rapaxane were 15, 30, 45, 60, 75, and 90 mg/kg, while the administered doses for Abraxane were 15, 30, 45, 30, 30, and 30 mg/kg. (b) Survival rates and (c) body weight changes of saline-, Rapaxane-, and Abraxane-treated groups were monitored over the course of the study for male and female mice. Complete blood count (CBC) results for neutrophil counts in (d) female and (e) male mice showed no severe neutropenia. Blue dashed lines represent the 5^th^ and 95^th^ percentiles of the saline group. Histopathological analysis at the study end point showed no significant toxicity in the kidneys, liver, lungs, spleen, or heart in either (f) female or (g) male mice treated with Rapaxane at 90 mg/kg compared to saline controls.

Complete blood count (CBC) results indicate that the administration of neither formulation at the designated dose caused severe neutropenia, as neutrophil counts remained within the normal range observed in saline-treated controls (5^th^ and 95^th^ percentiles, blue dashed line in Figure 6d,e for females and males, respectively). However, the impact of the formulations was more pronounced in reducing neutrophil counts in male mice (Figure 6e) compared to female mice (Figure 6d). Notably, survival rates were also lower in male mice compared to females (Figure 6b) in the Abraxane group at 45 mg/kg. Histopathological analysis at the study end point for Rapaxane treatment showed no significant differences compared to the saline group, with no major signs of toxicity observed in the kidneys, liver, lungs, spleen, or heart in either female (Figure 6f) or male (Figure 6g) mice.

One may question the observed toxicity of Abraxane despite its strong antitumor efficacy in the subcutaneous 4T1 breast cancer model. The toxicity profile of Abraxane following intravenous administration has been widely investigated across different mouse models. In immunodeficient mice, Desai et al.^69^ reported an MTD of 30 mg/kg when Abraxane was administered intravenously to female athymic NCr-nu nude mice on a qd × 5 schedule. When the dosing frequency was reduced, He et al.^70^ determined an MTD of 90 mg/kg in NCI-Nu/Nu mice administered every 4 days for a total of four injections (q4d × 4).

In immunocompetent mice, however, the toxicity of nanoparticle albumin-bound paclitaxel (nab-paclitaxel or Abraxane) can arise not only from paclitaxel as the active pharmaceutical ingredient but also from immunological reactions to the human serum albumin used in the formulation.^71^ Repeated i.v. injections of human albumin can elicit immune responses in mice, contributing to systemic toxicity. Several studies have reported widely varying MTD values for Abraxane depending on the mouse strain and dosing regimen. For example, Ernsting et al.^72^ found MTDs of 75 mg PTX/kg in NOD–SCID mice and 170 mg PTX/kg in C57BL/6 and BALB/c mice when administered as a single dose. Similarly, Neesse et al.^73^ reported an MTD of 120 mg/kg for Abraxane when given as a single i.v. dose to KPC mice.

To minimize the contribution of albumin-related immune responses and isolate PTX-associated toxicity, Neesse et al.^73^ also evaluated a murine albumin-bound paclitaxel (m-nab-paclitaxel) specifically formulated for preclinical use. In wild-type mice, they identified an MTD of 60 mg/kg administered every 3 days (q3d), with weight loss becoming apparent by day 15. This value exceeds the MTD observed in our study (30 mg/kg with repeated weekly dosing in BALB/c mice), supporting the conclusion that mortality at 45 mg/kg in our experiments may be partly attributed to immunological reactions to human albumin rather than to paclitaxel itself. Nonetheless, doses at or below 30 mg/kg were well tolerated, even with repeated administration.

### Rapaxane Demonstrates Superior Efficacy and Metastasis Prevention in an Orthotopic 4T1-*luc* Breast Cancer Mouse Model Compared to Abraxane and Single Prodrug-Loaded Formulations

We evaluated the efficacy of Rapaxane in inhibiting tumor growth and metastasis in an orthotopic 4T1-*luc* breast cancer mouse model. Orthotopic breast cancer models were established by injecting 4T1-*luc* cells into the mammary fat pad of female mice on day 14. Once tumor volumes reached approximately 100 mm^3^, the mice were randomly divided into six treatment groups: (i) saline, (ii) oLA_8_-PTX, (iii) oLA_8_-RAP, (iv) PTX/RAP, (v) Abraxane, and (vi) Rapaxane. Formulations were administered via intravenous tail vein injections at doses of 10 mg/kg PTX equivalent for PTX or oLA_8_-PTX and 2 mg/kg RAP equivalent for RAP or oLA_8_-RAP on days 0, 7, and 14 (Figure 7a–b).

**Figure 7:**
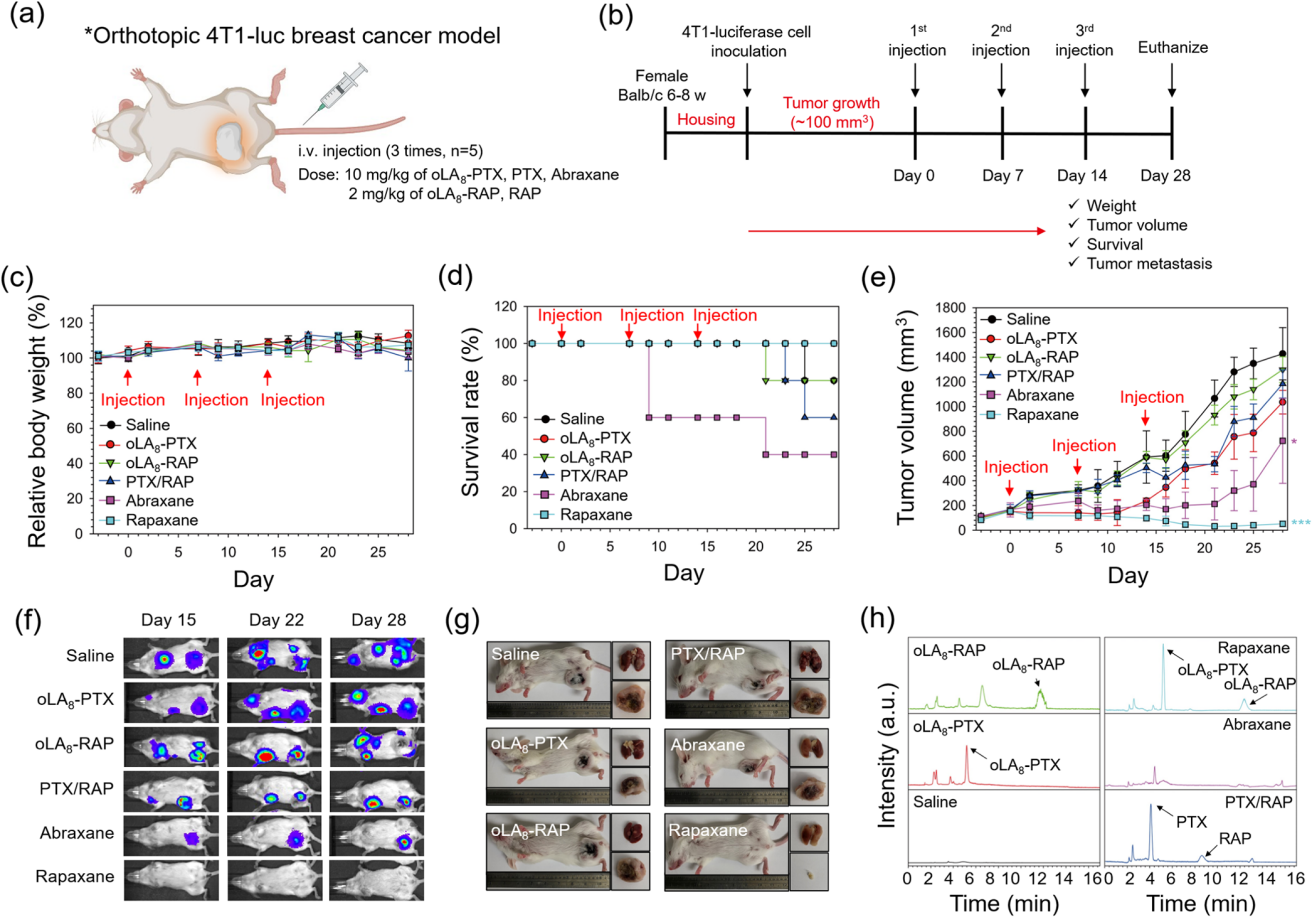
Therapeutic efficacy of Rapaxane in an orthotopic 4T1 breast cancer model. (a) Establishment of the orthotopic 4T1 breast cancer model. (b) Experimental design and treatment schedule for the orthotopic 4T1 breast cancer model. (c) Relative changes in body weight and (d) survival rate of the tumor-bearing mice after treatment with saline (control), oLA_8_-PTX, oLA_8_-RAP, PTX/RAP, Abraxane, and Rapaxane (i.v. injections administered on days 0, 7, and 14). Mice were weighed every 2–3 days over 28 days (*n* = 5 per group; doses: 10 mg/kg PTX equivalent; 2 mg/kg RAP equivalent). (e) Tumor volume variations in orthotopic 4T1-*luc* breast cancer models after treatment. Data represent means ± S.E.M., with *p ≤ 0.05 and ***p ≤ 0.001 compared to the saline group. (f) Representative bioluminescence IVIS images of 4T1-*luc* cells on days 15, 22, and 28, captured 10 min after D-luciferin injection. (g) Representative photographs of the tumor-bearing mice (left), harvested lungs (top-right), and harvested tumors (bottom-right) on day 28. (h) Representative RP-HPLC chromatograms of tumor extracts harvested 2 h after i.v. injections of saline, oLA_8_-PTX, oLA_8_-RAP, PTX/RAP, Abraxane, and Rapaxane, following tumor homogenization and acetonitrile extraction.

The lower doses used in this study, compared to the subcutaneous model, as well as the selection of control groups, were aimed at accounting for the reduced tolerance to Abraxane in the orthotopic model. Initially, we considered Abraxane + oral everolimus (EVE) as a potential control. We tested 30 mg/kg of Abraxane, which was well tolerated in healthy mice, in combination with oral EVE (6 mg/kg twice a week) in the orthotopic model; however, this combination proved to be toxic and lethal (Figure S9). Additionally, administering 30 mg/kg of Abraxane alone in the orthotopic model resulted in severe acute toxicity and mortality (data not shown), despite being tolerated in healthy mice. Consequently, we selected 10 mg/kg Abraxane without EVE as the control group.

Throughout the experiment, body weight, survival rate, and tumor volume were monitored (Figure 7c–e). Body weight remained stable across all groups over 28 days (Figure 7c). Consistent with results from the subcutaneous tumor model, the oLA_8_-PTX and Rapaxane-treated groups showed a significantly higher survival rate (100%) compared to oLA_8_-RAP (80%), PTX/RAP (60%), and Abraxane (40%) (Figure 7d). The survival results underscore the superior safety of Rapaxane.

Following three weekly injections, tumor volume was significantly reduced in the Rapaxane-treated group to 51 ± 24 mm^3^, compared to saline (1428 ± 211 mm^3^), oLA_8_-PTX (1036 ± 95 mm^3^), oLA_8_-RAP (1299 ± 104 mm^3^), PTX/RAP (1184 ± 114 mm^3^), and Abraxane (723 ± 344 mm^3^) (Figure 7e). These results highlight Rapaxane’s potential for significantly improving therapeutic outcomes in TNBC through superior tumor growth inhibition and survival benefits. Considering the incomplete survival observed in the oLA_8_-RAP, PTX/RAP, and Abraxane treatment groups, it should be noted that tumor volumes for these groups were calculated based on the average tumor size of the surviving animals at each time point, and all statistical analyses were performed accordingly.

We also evaluated tumor metastasis by measuring the bioluminescence signal generated by 4T1-*luc* cells (Figure 7f). On days 15, 22, and 28, the mice were injected with 100 μL of d-luciferin and imaged using IVIS 10 min post injection. The IVIS images showed strong bioluminescence signals in both the tumor and lungs for the saline, oLA_8_-PTX, oLA_8_-RAP, and PTX/RAP treatment groups. However, the absence of bioluminescence signals in the lungs for both Abraxane and Rapaxane-treated mice suggests significant inhibition of metastasis by these formulations compared to the others. In the case of Rapaxane, the bioluminescence signal from the tumor was also too weak to be detected under the adjusted exposure settings, indicating a substantially smaller tumor size. This observation aligns with the tumor volume measurements (Figure 7e) and the harvested tumor sizes on day 28 (Figure 7g). The extent of metastasis was also quantified based on the intensity of the bioluminescence signal for each group, as shown in Figure S10.

RP-HPLC analysis of tumor samples collected 2 h post injection revealed the presence of intact oLA_8_-PTX and oLA_8_-RAP in Rapaxane-treated groups (Figure 7h). This observation likely reflects the stability of the formulation during the early post administration period, as previous studies have shown that the free prodrugs rapidly undergo conversion to smaller species within minutes of plasma exposure.^46^ However, this observation cannot be extrapolated to later time points as the potential accumulation of partially hydrolyzed o­(LA)_*n*_-PTX and o­(LA)_*n*_-RAP species derived from plasma conversion remains undetermined. A comprehensive PK and biodistribution study covering extended time points and including all potential species is required to draw definitive conclusions regarding the long-term stability and distribution of the prodrugs.

Next, we used H&E staining of the lungs and tumors to investigate metastasis to the lungs. The H&E staining images (Figure 8a) reveal dense clusters of metastatic tumor cells in the saline-treated group, indicating extensive lung metastasis. Evidence of metastasis was also observed in the oLA_8_-PTX, oLA_8_-RAP, and PTX/RAP groups, as demonstrated by dense clusters of tumor cells compressing or displacing the alveoli, resulting in the loss of normal lung architecture.

**Figure 8:**
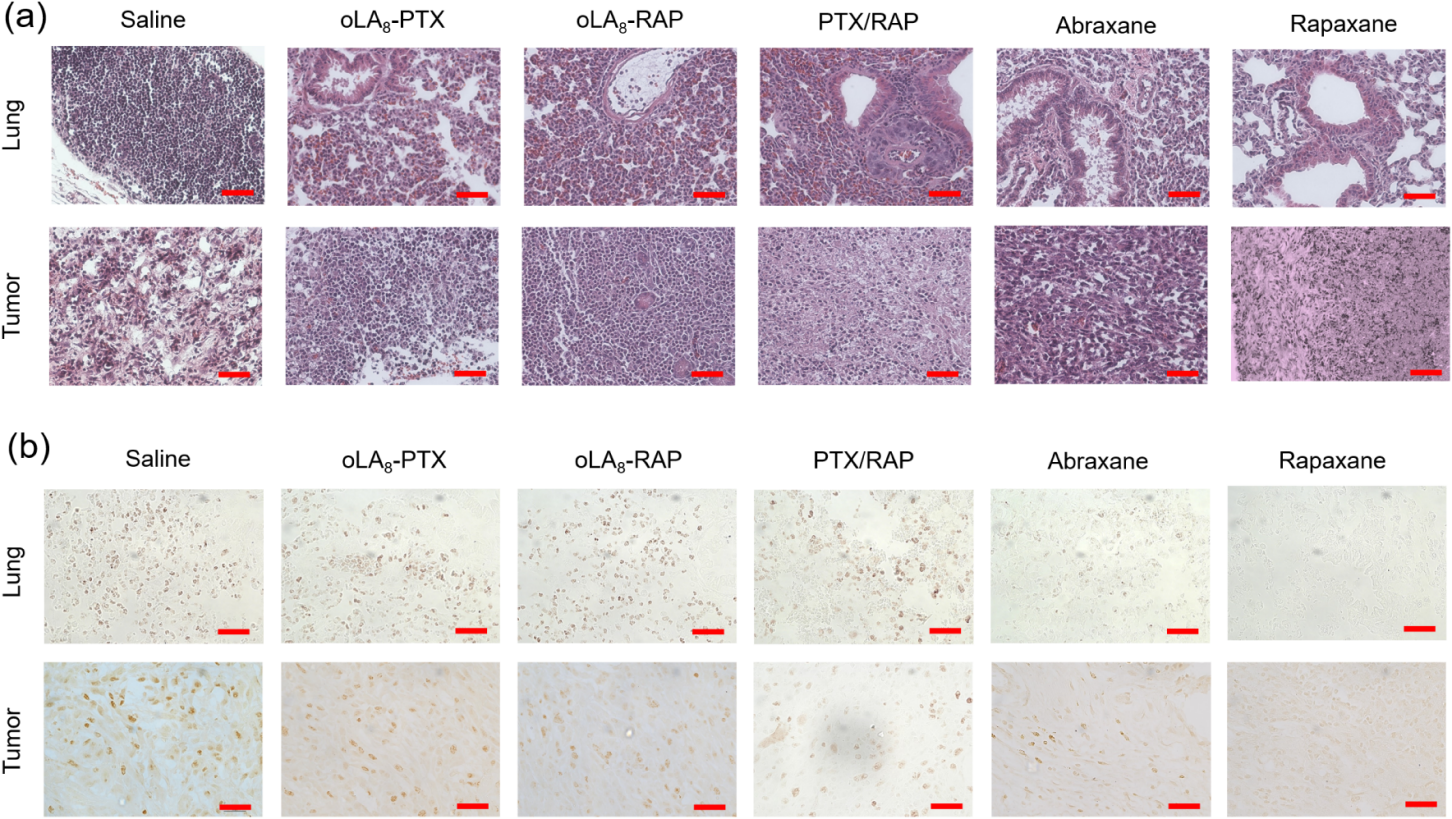
Histological analysis of orthotopic 4T1 breast cancer model mice. (a) H&E staining of lung and tumor sections after treatment with saline, oLA_8_-PTX, oLA_8_-RAP, PTX/RAP, Abraxane, and Rapaxane (i.v. injection, 3 times). (b) Ki-67 IHC images of paraffin-embedded tumor sections. Scale bars: 100 μm.

In contrast, lung tissue from the Abraxane and Rapaxane-treated groups displayed a more preserved morphology, dominated by thin-walled alveoli lined by squamous epithelium. The Rapaxane-treated group, in particular, exhibited a healthier lung morphology with near-normal tissue architecture and reduced signs of tumor invasion compared to the Abraxane-treated group.

We also performed immunohistochemical (IHC) staining for Ki-67, a marker of cell proliferation, in both tumor and lung tissues (Figure 8b). The results revealed that cell proliferation was markedly reduced in the Rapaxane-treated group compared to other treatment groups and the saline control. In lung tissue, both the Rapaxane and Abraxane groups exhibited the lowest Ki-67 expression, indicating significantly reduced cell proliferation, likely due to reduced or inhibited metastasis. The saline group showed the highest Ki-67 signal, reflecting enhanced proliferation activity in metastatic lesions, which is consistent with the IVIS and H&E data. The PTX/RAP, oLA_8_-RAP, and oLA_8_-PTX groups displayed intermediate levels of Ki-67, suggesting partial inhibition of the proliferation. In tumor tissue, Rapaxane showed the lowest Ki-67 staining, indicating a significant antiproliferative effect against tumor cells. The saline group exhibited the highest Ki-67 staining, as expected. The PTX/RAP group also demonstrated a notable decrease in Ki-67 expression, though not as pronounced as Rapaxane. The oLA_8_-RAP, Abraxane, and the oLA_8_-PTX groups exhibited intermediate levels of Ki-67-positive cells, with variable staining intensity. Overall, these results demonstrate that combination therapy using Rapaxane is highly effective in preventing lung metastasis and preserving normal tissue architecture compared to all other treatments. This underscores its potential as a superior therapeutic option for targeting triple-negative breast cancer and its associated metastases.

Combining agents with complementary mechanisms of action provides more effective control of malignant growth.^74–77^ Rationally designed drug combinations can exploit mechanisms such as cell-cycle regulation, redox imbalance, and angiogenic signaling to maximize tumor suppression while minimizing systemic toxicity.^78–82^ Moreover, combination regimens can overcome multidrug resistance (MDR), a major obstacle in cancer therapy, by targeting multiple resistance mechanisms, including P-glycoprotein-mediated efflux and altered apoptotic pathways.^83^ In advanced disease stages, combinatorial approaches that integrate immunomodulators or checkpoint inhibitors further enhance immune recognition and elimination of metastatic or residual cancer cells.^84,85^

One notable clinical example of combination therapy using nanotechnology is Vyxeos, a dual-drug liposomal formulation containing daunorubicin (DNR) and cytarabine (Ara-C) at a fixed 1:5 molar ratio approved for the treatment of acute myeloid leukemia. Vyxeos demonstrated a prolonged half-life, a six-week therapeutic effect, and selective drug uptake, 2–9 fold higher in leukemia cells than in normal bone marrow, establishing the clinical significance of fixed-ratio nanocarrier codelivery.^78–80^

Building on this rationale, the present study introduces Rapaxane, a fixed-ratio nanocarrier codelivering paclitaxel and rapamycin. While PTX/RAP codelivery was previously explored by Blanco et al.,^68^ Rapaxane differs fundamentally in several key aspects, including the optimized PTX/RAP ratio, the observation of synergy at lower concentrations, the use of oligo­(lactic acid) prodrugs rather than free drugs, and a sustained drug release profile that extends systemic exposure. The prodrug-based design of Rapaxane enables higher tolerated doses and improved safety compared to Abraxane and conventional PTX/RAP formulations.^49^

While nanocarriers have been extensively utilized for the codelivery of drug combinations, most reported formulations employ parent drugs^86–92^ that differ significantly in physicochemical properties, release kinetics, and PK profiles. These disparities often hinder efficient coencapsulation, synchronized release, and ratiometric exposure in vivo. The use of prodrugs provides a powerful strategy to tune such properties, improving compatibility, stability, and controlled release. For instance, Li et al.^93^ developed a cascade-responsive prodrug nanoplatform for optimized multidrug delivery, employing redox-responsive prodrugs of paclitaxel, doxorubicin, and camptothecin assembled with a poly­(lactide)-poly­(*N*-(2-hydroxypropyl)­methacrylamide) copolymer to bridge the gap between in vitro combination screening and in vivo therapeutic efficacy. However, such systems rely heavily on maintaining nanoparticle integrity to ensure the complete delivery and synchronized release of the active drugs. In contrast, the strategy used to design Rapaxane mitigates issues arising from mismatched PK profiles and reduces the dependence on nanoparticle integrity for ratiometric drug delivery, thereby offering a more reliable and translationally robust approach to combination chemotherapy.

It should be noted that beyond nanocarriers, antibody–drug conjugates (ADCs) represent another precision-driven approach to combination therapy. ADCs couple tumor-targeting antibodies with potent cytotoxic payloads via cleavable linkers, ensuring selective drug release at the tumor site. For example, RC48 (HER2-ADC) exhibits enhanced specificity and safety compared to conventional HER2-targeted therapies, while advances in linker chemistry continue to minimize off-target effects.^94,95^

In brief, the data presented suggest that o­(LA)_n_-prodrugs coencapsulated in PEG-*b*-PLA polymeric micelles enable ratiometric loading, release, and conversion to the parent drugs. By employing a synergistic ratio of such prodrugs in the formulation, Rapaxane has been developed as a promising therapeutic option capable of inhibiting tumor growth and metastasis while maintaining an encouraging safety profile. Rapaxane demonstrates significant potential as a superior treatment for triple-negative breast cancer and its associated metastases. Moving forward, we plan to conduct further in-depth characterization of Rapaxane, including pharmacokinetic studies to assess ratiometric exposure and biodistribution studies to evaluate ratiometric accumulation in the tumor, with the ultimate goal of advancing the development of Rapaxane.

## Conclusion

This study highlights the potential of Rapaxane, a ratiometric drug delivery system consisting of PEG-*b*-PLA micelles coloaded with oligo­(lactic acid)_8_ conjugated prodrugs of paclitaxel (oLA_8_-PTX) and rapamycin (oLA_8_-RAP), for treating triple-negative breast cancer. Rapaxane achieves high encapsulation efficiency, synchronized release, and precise maintenance of the synergistic 5:1 drug ratio, addressing the pharmacokinetic and toxicity limitations associated with conventional combination therapies.

In vitro studies demonstrated that Rapaxane exhibits remarkable cytotoxicity against TNBC cell lines compared with single prodrug treatments. In vivo, Rapaxane significantly outperformed Abraxane and other treatment groups in subcutaneous and orthotopic TNBC mouse models. Key findings include significant tumor volume reduction, inhibition of metastasis, and improved survival rates. Rapaxane’s dual functionality in tumor growth inhibition and metastasis prevention highlights its promise as an advanced therapy for TNBC.

## Methods

General information regarding reagents and analytical methods used in this study is available in the Supporting Information.

### Determination of the Optimal Ratio of oLA_8_-PTX:oLA_8_-RAP

To determine the optimal ratio of oLA_8_-PTX and oLA_8_-RAP, 4T1 and MDA-MB-231 cells (3 × 10^4^ cells/mL, 100 μL) were seeded into 96-well plates and incubated for 24 h at 37 °C. The cells were then treated with oLA_8_-PTX and oLA_8_-RAP in combination ratios ranging from 5:1 to 1:5 and further incubated for 72 h at 37 °C. Cell viability was assessed using the CellTiter-Blue assay to determine IC50 values. Combination index (CI) values, which classify interactions as antagonistic (CI > 1), additive (CI = 1), or synergistic (CI < 1), were calculated at different fractions of affected cells using CompuSyn software, based on the median-effect Chou and Talalay equation^53^ to determine the optimal ratio of PTX and RAP (Supporting Information).

### Preparation of oLA_8_-PTX/oLA_8_-RAP Coloaded Micelles

Coloaded micelles with various ratios of oLA_8_-PTX and oLA_8_-RAP were prepared using the PEG-assist method, a recently developed protocol in our lab.^50^ Prodrugs were dissolved with PEG_4 kDa_-*b*-PLA_2.2 kDa_ and PEG2000 (average Mw: 2000) at a 1:1:20 weight ratio of prodrug: PEG_4 kDa_-*b*-PLA_2.2 kDa_:PEG2000 at 70 °C with gentle vortex mixing. Once a clear solution was achieved, it was equilibrated at 60 °C for 1 h. Prewarmed Milli-Q water (60 °C) was added to the solution, followed by brief vortexing and a 1 h equilibration at room temperature (25 °C). The solutions were centrifuged at 10000g for 5 min to remove unencapsulated prodrugs.

### Characterization of oLA_8_-PTX/oLA_8_-RAP Coloaded Micelles

The hydrodynamic size of the micelles was measured using dynamic light scattering (DLS) with a Malvern Instruments Zetasizer Nano ZS90 instrument (Worcestershire, UK). Measurements were performed at 25 °C with a detection angle of 173° and a He–Ne ion laser light source (4 mW, 633 nm). Samples were diluted to a PEG_4 kDa_-*b*-PLA_2.2 kDa_ concentration of ∼0.1 mg/mL in an aqueous solution and measured in disposable sizing cuvettes.^96^ Encapsulation efficiency and weight-loading percentages (loading capacity) were determined using reversed-phase high-performance liquid chromatography (RP-HPLC) with a Shimadzu Prominence system (Kyoto, Japan) equipped with an LC-20AT pump, SIL-20AC HT autosampler, CTO-20AC column oven, and SPD-M20A diode array detector. Samples (10 μL) were injected onto a Zorbax RX-C8 column (5 μm, 4.6 × 250 mm; Agilent Technologies, Santa Clara, CA, USA) and analyzed under isocratic conditions (75% acetonitrile (ACN) and 25% Milli-Q water) at 40 °C with a 1 mL/min flow rate. Samples were diluted in 100% ACN before injection. oLA_8_-PTX was monitored at 227 nm, and oLA_8_-RAP at 279 nm.^46,47^ Encapsulation efficiency was calculated using the following equations:^97^

$$\mathrm{Encapsulation}\;\mathrm{efficiency}\;\left(\%\right)=\frac{\mathrm{Drug}\;\mathrm{loaded}\;\mathrm{in}\;\mathrm{micelles}\;\left(g\right)}{\mathrm{Initially}\;\mathrm{added}\;\mathrm{drug}\;\left(g\right)}\times 100$$ 1

$$\mathrm{Weight}\;\mathrm{loading}\;\mathrm{capacity}\;\left(\%\right)=\frac{\mathrm{Drug}\;\mathrm{loaded}\;\mathrm{in}\;\mathrm{micelles}\;\left(g\right)}{\mathrm{Total}\;\mathrm{loaded}\;\mathrm{drug}+\mathrm{Copolymer}\;\left(g\right)}\times 100$$ 2

Prodrug release from PEG_4 kDa_-*b*-PLA_2.2 kDa_ micelles was evaluated in PBS (pH 7.4) under sink condition.^46^ Prodrug-loaded micelles (0.5 mg/mL, PBS, pH 7.4) were added to 20 kDa molecular weight cutoff (MWCO) Slide-A-Lyzer dialysis cassettes (Thermo Scientific, Waltham, MA, USA) and dialyzed against 4 L PBS at 37 °C with constant stirring. At specific intervals, 100 μL samples were collected and replaced with fresh PBS. Samples were diluted with 900 μL of ACN and analyzed by RP-HPLC.

For prodrug conversion analysis, micelles encapsulating prodrugs (1 mg/mL, PBS, pH 7.4) were incubated at 37 °C with gentle shaking. At designated time points, 50 μL samples were collected and diluted with 950 μL of ACN for RP-HPLC analysis to quantify converted prodrug species. All metabolites of oLA_8_-PTX and oLA_8_-RAP, containing different numbers of lactic acid oligomers ranging from 0 to 7, were analyzed using gradient mode RP-HPLC. The organic phase consisted of 100% acetonitrile (solvent A), while the aqueous phase was 100% Milli-Q water (solvent B). The gradient elution was programmed as follows: starting at 50% solvent A and 50% solvent B at 0 min, transitioning to 95% solvent A and 5% solvent B over 35 min, followed by a 40-min equilibration phase.

### Animals

All animal experiments were conducted under protocol ID: M005844, approved by the School of Medicine and Public Health Institutional Animal Care and Use Committee at the University of Wisconsin–Madison, in accordance with institutional and NIH guidelines for the Care and Use of Laboratory Animals.

Six- to eight-week-old BALB/c mice were procured from Jackson Laboratory (Bar Harbor, ME, USA). The mice were housed in groups of five per cage with corn cob bedding. The housing conditions were maintained at 23 ± 1 °C with 60 ± 10% relative humidity on a 12-h light/dark cycle.

### Therapeutic Efficacy in Subcutaneous 4T1 Breast Cancer Model

To establish a subcutaneous 4T1 breast cancer model, 4T1 cells (1 × 10^6^ cells suspended in 100 μL of PBS) were injected subcutaneously behind the flank of each mouse under isoflurane anesthesia. Following tumor inoculation, mice were allowed to recover in a temperature-controlled chamber before being housed individually. Two weeks post implantation (average tumor volume ≈ 100 mm^3^), mice were randomly assigned to six groups (*n* = 5 per group): (i) saline (control), (ii) oLA_8_-PTX-loaded micelle, (iii) oLA_8_-RAP-loaded micelle, (iv) PTX/RAP coloaded micelle (5:1 PTX to RAP ratio), (v) Abraxane, and (vi) Rapaxane (oLA_8_-PTX and oLA_8_-RAP coloaded micelle, 5:1 ratio). The treatment doses were 50 mg/kg paclitaxel (PTX or oLA_8_-PTX) and 10 mg/kg rapamycin (RAP or oLA_8_-RAP) for relevant formulations. Treatments were administered on Days 0, 7, and 14 via intravenous injection. Tumor volume was measured using a caliper and calculated as *V* = (4/3) × π × (*L*/2)^2^ × (*W*/2), where *L* is the length and *W* is the width of the tumor.^97^

### Assessment of oLA_8_-PTX/oLA_8_-RAP Ratio in Blood

To confirm the maintenance of the oLA_8_-PTX/oLA_8_-RAP ratio in plasma, blood samples were collected from the retro-orbital vein of mice bearing subcutaneous 4T1 breast cancer tumors 5 min after the first tail vein injection and subsequently analyzed. The blood samples, collected under isoflurane anesthesia, included all six treatment groups: saline, oLA_8_-PTX (50 mg/kg), oLA_8_-RAP (10 mg/kg), PTX/RAP (PTX: 50 mg/kg, RAP: 10 mg/kg), Abraxane (50 mg/kg), or Rapaxane (oLA_8_-PTX: 50 mg/kg, oLA_8_-RAP: 10 mg/kg). Samples were transferred to 0.5 mL BD Minicollect tubes pretreated with K2EDTA (Greiner Bio-One, Kremsmünster, NJ, USA) and kept on ice. Plasma was separated by centrifugation at 3000 rpm for 3 min at 4 °C and then stored at −80 °C until analysis. Drug and prodrug concentrations in plasma were quantified by using RP-HPLC.

### Toxicity Study

A dose titration study was conducted to determine the maximum tolerated dose (MTD) of Rapaxane and Abraxane. The study commenced with an initial dose of 15 mg/kg PTX-equivalent, which was escalated to 15 mg/kg weekly. The total duration of the study was 8 weeks, consisting of 4 weeks of treatment, followed by 2 weeks of rest and then an additional 2 weeks of treatment.

During the study, mice were monitored for survival and body weight changes. Hematological assessments, including blood counts, were performed twice a week to screen for neutropenia. Blood was collected via retro-orbital bleeding once 1 day post injection and again 3 days postinjection for both Abraxane and Rapaxane.

At the conclusion of the study, mice treated with Rapaxane at 90 mg/kg were euthanized for histopathological evaluation. Formalin-fixed tissues were subjected to hematoxylin and eosin (H&E) staining to assess potential toxicological effects, with comparisons made to a saline-treated control group.

### Therapeutic Efficacy in Orthotopic 4T1-*luc* Breast Cancer Model

Orthotopic 4T1-*luc* breast cancer model mice were established by injecting 4T1-*luc* cells (1 × 10^5^ cells suspended in 50 μL PBS) into the mammary fat pad under isoflurane anesthesia.^96^ Two weeks post-transplantation (average tumor volume ≈ 100 mm^3^), mice were randomly divided into six groups (*n* = 5 per group) as detailed above for subcutaneous studies. Treatment doses were 10 mg/kg of paclitaxel (PTX or oLA_8_-PTX) and 2 mg/kg of rapamycin (RAP or oLA_8_-RAP), administered intravenously once a week for three weeks. Body weight and tumor volume were monitored every 2–3 days. On Day 28, mice were euthanized via CO_2_ asphyxiation, per AVMA guidelines. Tumor and lung tissues were harvested for immunohistochemical (IHC) staining and hematoxylin and eosin (H&E) staining.

### Statistical Analysis

Data were analyzed using one-way ANOVA for multiple comparisons, followed by Tukey’s post hoc test (SigmaPlot, Systat Software, Inc.). Statistical significance was set at *p* < 0.05, unless otherwise noted.

## Supplementary Material



## Notes

Materials and methods for synthesis and characterization of oLA_8_-PTX and oLA_8_-RAP; procedures for in vitro cytotoxicity assays, hemolysis evaluation, metastasis quantification, histopathology, and immunohistochemistry (IHC); ^1^H NMR and LC-MS spectra of oLA_8_-PTX and oLA_8_-RAP; cell viability curves, IC50 values, and combination index (CI) analysis for PTX/RAP and oLA_8_-PTX/oLA_8_-RAP combinations; physicochemical characterization and colloidal stability of prodrug-loaded micelles in various media; reconstituted micelle characterization following lyophilization; hemolysis profiles of Rapaxane versus comparator formulations; evaluation of Rapaxane vs Abraxane + oral Everolimus in the orthotopic breast cancer model; bioluminescence-based quantification of lung metastasis (PDF)
