# Supplementary material for: Precise Ratiometric Drug Delivery for the Treatment of Triple-Negative Breast Cancer
Source: ACS Nano. 2025 Nov 19;19(47):40456–72. doi: 10.1021/acsnano.5c13083 (PMC12676740; doi:10.1021/acsnano.5c13083)
Supplement: Supplementary file 1 [file nn5c13083_si_001.pdf]

Supporting Information

Precise ratiometric drug delivery for the treatment of triple-negative breast cancer

Rae Hyung Kang [a,b,†], Morteza Rasoulianboroujeni [a,c,†], Maryam Kianpour [a], Lauren Repp [a], Suzanne M. Ponik [d], Glen S. Kwon [a,*]

[a] Pharmaceutical Sciences Division, School of Pharmacy, University of Wisconsin-Madison, Madison, WI 53705, USA

[b] Department of Pharmaceutical Engineering, Dankook University, Cheonan 31116, Republic of Korea

[c] Department of Pharmaceutical Sciences, Gatton College of Pharmacy, East Tennessee State University, Johnson City, TN 37614, USA

[d] Department of Cell and Regenerative Biology, School of Medicine and Public Health, University of Wisconsin-Madison, Madison, WI 53705, USA

[†] Authors contributed equally

[*] Corresponding author: glen.kwon@wisc.edu

This file includes:

1. Materials and Methods
2. Supporting Figures: S1 to S10
3. Supporting Tables: S1 to S9
4. References

1. Materials and Methods

1.1. General Information

Paclitaxel (PTX, product No. P-9600) and Rapamycin (RAP, product No. R-5000) were purchased from LC Laboratories (Woburn, MA, USA). TBS-o(LA)$_8$ was purchased from Proactive Molecular Research (Gainesville, FL, USA). Poly(ethylene glycol)-*block*-poly(lactic acid) (PEG-*b*-PLA) with average PEG Mn of 4 kDa and PLA Mn of 2.2 kDa was purchased from JenKem USA (Plano, TX, USA). Poly(ethylene glycol) (PEG, average Mw. 2,000, Product No. 84797) and acetonitrile (ACN, product No. 34851) were purchased from Sigma-Aldrich (St. Louis, MO, USA). Abraxane (product No. NC2210238) was purchased from Selleck Chemical Llc. (Houston, TX, USA). 4T1 and MDA-MB-231 breast cancer cells were purchased from ATCC (Manassas, VA). Dulbecco's Modification of Eagles Medium (DMEM, Product No. 10-013-CV), Phosphate-Buffered Saline (1X PBS, pH 7.4, product No. 45000-448), and Matrigel (product No. CB354248) were purchased from Corning (Corning, NY, USA). Fetal Bovine Serum (FBS, product No. 10082147) was purchased from Thermo Fisher Scientific (Waltham, MA, USA). Trypsin-EDTA (0.25%, product No. 25200056) and Penicillin-streptomycin (product No. 15140-122) were purchased from Gibco (Billings, MT, USA). Cell Titer-Blue cell viability assay kit (product No. G8081) was purchased from Promega Corp. (Madison, WI, USA). Paraformaldehyde solution (4% in PBS, product No. Sc-281692) was purchased from Santa Cruz Biotechnology (Dallas, TX, USA). CD31 (PECAM-1) antibody (product No. 77699T) was purchased from Cell Signaling Technology (Danvers, MA, USA). Commercially available reagents and solvents were used without further purification.

1.2. Determination of the optimal ratio of PTX and RAP

The cytotoxicity of PTX, RAP, and their combinations (5:1 to 1:5 ratio) was investigated against 4T1 murine and MDA-MB-231 human breast cancer cells using the Cell Titer Blue Cell Viability Assay (Promega; Madison, WI, USA). Cells (3 × 10^3 cells/well) were seeded on 96-well plates and incubated in Dulbecco's Modified Eagle Medium (DMEM) supplemented with 10% fetal bovine serum and 1% penicillin-streptomycin for 24 h at 37 °C in 5% CO_2. After 24 h incubation, the cells were treated with drugs and their combination (0−100 μM) and further incubated for 72 h. The cytotoxicity of the drugs was evaluated using a Cell Titer Blue assay kit following the manufacturer's instructions. The fluorescent intensity of viable cells was recorded by a SpectraMax M2 plate reader (Molecular Devices; San Jose, CA, USA) with 560 nm excitation and 590 nm emission. The inhibitory concentration 50 (IC50) was calculated using GraphPad Prism software (San Diego, CA, USA). Combination index (CI) values were determined with the Chou and Talalay method and CompuSyn software based on the following equation:

$$CI = (D_{c1}/D_{s1}) + (D_{c2}/D_{s2})$$

where D_{c1} and D_{c2} represent the IC50 values of the drugs applied in combination and D_{s1} and D_{s2} represent the IC50 values of the drugs individually. The combination index was calculated at different fractions of affected cells (Fa) where Fa = 0 represents 100% cell viability and Fa = 1 represents 0% cell viability. The effects of the combination are described as follows: CI < 0.9, synergism; CI = 0.9–1.1, additive; CI > 1.1, antagonism.

1.3. Preparation of oLA$_8$-PTX and oLA$_8$-RAP

The o(LA)$_8$-PTX and o(LA)$_8$-RAP prodrugs were synthesized using our previously reported procedures [1, 2]. The structures, molecular masses, and purities were confirmed using proton nuclear magnetic resonance (^{1}H NMR) and liquid chromatography-mass spectrometry (LC-MS). NMR was conducted using a Varian Unity-Inova three-channel 500 MHz spectrometer (Varian Inc, Palo alto, CA). The temperature was set to 25 °C and chemical shifts (d) were reported in parts per million relative to residual protonated chloroform-d ($CDCl_3$) resonance at 7.26 ppm. ^{1}H NMR of o(LA)$_8$-PTX (500 MHz, $CDCl_3$): d = 8.17 - 7.31 (m, 15 H), 7.01 (d, J = 8.9 Hz, 1 H), 6.29 (s, 1 H), 6.23 - 6.13 (m, 1 H), 5.85 - 5.75 (m, 1 H), 5.68 (d, J = 7.0 Hz, 1 H), 5.62 - 5.47 (m, 1 H), 5.33 - 5.02 (m, 7 H), 4.91 (d, J = 8.5 Hz, 1 H), 4.79 (dd, J = 2.5, 4.9 Hz, 1 H), 4.38 - 4.33 (m, 1 H), 4.32 (d, J = 8.3 Hz, 1 H), 4.19 (d, J = 7.8 Hz, 1 H), 3.92 (d, J = 6.8 Hz, 1 H), 3.54 (d, J = 4.9 Hz, 1 H), 2.67 - 2.55 (m, 2 H), 2.38 (s, 3 H), 2.33 (d, J = 8.1 Hz, 1 H), 2.15 (s, 3 H), 1.84 (s, 4 H), 1.81 (s, 3 H), 1.64 - 1.46 (m, 24 H), 1.20 (s, 3 H), 1.16 (s, 3 H). ^{1}H NMR of o(LA)$_8$-RAP (400 MHz, $CDCl_3$): δ 6.38 (dd, J = 10.5, 14.5 Hz, 1H), 6.31 (dd, J =

10.0, 15.0 Hz, 1H), 6.14 (dd, J = 10.0, 15.0 Hz, 1H), 5.96 (d, J = 11.0 Hz, 1H), 5.54 (dd, J = 9.0, 15.0 Hz, 1H), 5.41 (d, J = 9.5 Hz, 1H), 5.28 (d, J = 6.5 Hz, 1H), 5.24–5.11 (m, 8H), 4.77 (s, 1H), 4.71 (ddd, J = 5.0, 9.0, 11.5 Hz, 1H), 4.18 (d, J = 6.0 Hz, 1H), 3.74 (d, J = 5.5 Hz, 1H), 3.67 (q, J = 7.0 Hz, 1H), 3.57 (d, J = 20.5 Hz), 3.37 (s, 3H), 3.33 (s, 3H), 3.14 (s, 3H), 2.71 (dd, J = 5.5, 17.0 Hz, 1H), 2.66 (d, J = 6.0 Hz, 1H), 2.57 (dd, J = 5.5, 17.0 Hz, 1H), 1.09 (d, J = 6.5 Hz, 3H), 1.05 (d, J = 7.0 Hz, 3H), 0.99 (d, J = 6.5 Hz, 3H), 0.95 (d, J = 6.5 Hz, 3H), 0.90 (d, J = 7.0 Hz, 3H). LC-MS was conducted with an Agilent 1290 Infinity II equipped with a 6120 single quadrupole MS (Agilent Technologies; Santa Clara, CA, USA). A gradient method consisting of 5% methanol (MeOH) in H_2O with 0.1% formic acid (A) and MeOH with 0.1% formic acid (B) was used. Spectra are included in the supplementary materials.

1.4. Cytotoxicity of various ratios of oLA_8-PTX/oLA_8-RAP co-loaded micelle

The cytotoxicity of micelles loaded with various ratios of oLA_8-PTX/oLA_8-RAP against 4T1 and MDA-MB-231 cells was analyzed using the same methods with free drug and free oLA_8-PTX/oLA_8-RAP. In brief, the cells (100 µL, 3×10^4 cells/mL) were seeded on the 96-well plated and incubated for 24 h at 37 °C. The cells were treated with micelles with pre-calculated prodrug concentration (0−100 µM) and further incubated for 72 h. The cytotoxicity was measured using the Cell Titer Blue and IC50 values were calculated using GraphPad Prism software (San Diego, CA, USA).

1.5. Hemolysis analysis of Rapaxane

To evaluate the hemolytic properties of Rapaxane, blood samples were collected from the hearts of mice under isoflurane anesthesia. The samples were centrifuged at 3,000 rpm for 3 min at 4 °C after washing with cold 1x PBS (pH 7.4) to isolate red blood cells (RBCs). The RBC suspension (8% v/v) was incubated for 1 hour at 37 °C with 0.1 mg/mL of oLA_8-PTX-loaded micelle (oLA_8-PTX), oLA_8-RAP-loaded micelle (oLA_8-RAP), PTX/RAP co-loaded micelle (PTX/RAP), Abraxane®, Rapaxane, and 0.1% (v/v) Triton X-100 (positive control). Following incubation, samples were centrifuged at 3,000 rpm for 3 min at 4 °C. The absorbance of the supernatant was measured at 492 nm using a microplate reader at 25 °C to assess hemolysis [3].

1.6. Tumor metastasis Analysis

IVIS imaging was performed to confirm the tumor metastasis of 4T1-luc in orthotopic 4T1-luc breast tumor model mice. The randomly divided tumor-bearing mice to 5 groups (n = 5) as following; (i) control group (i.v. injected with saline), (ii) oLA_8-PTX group (i.v. injected with oLA_8-PTX loaded micelle), (iii) oLA_8-RAP group (i.v. injected with oLA_8-RAP loaded micelle), (iv) PTX/RAP (i.v. injected with 5:1 ratio of PTX and RAP co-loaded micelle), (v) Abraxane (i.v. injected with Abraxane), and (vi) Rapaxane (i.v. injected with 5:1 ratio of oLA_8-PTX and oLA_8-RAP co-loaded micelle) were intraperitoneally injected with luciferase (100 µL, 0.1 mg/mL) and circulated for 10 min on day 15, 22, and 28. The luminescence signals from the whole body of mice were acquired using the In Vivo Imaging System (IVIS, Perkin Elmer, Waltham, MA, USA).

1.7. Histology analysis (H&E staining)

After the mice were sacrificed, the lung and tumor tissue were fixed by vascular perfusion using a 4% paraformaldehyde fixative solution after all blood was removed using PBS perfusion on day 28. Excreted tissues were then fixed in formaldehyde, rinsed under running tap water, and embedded in paraffin wax. Sections of paraffin blocks were sliced into 4–5 µm and transferred to a slide. The sliced tissue sections were deparaffinized in xylene and rehydrated in absolute ethanol series (100%, 90%, 80%, and 70%). For Hematoxylin and eosin (H&E) staining, the sliced sections were stained in hematoxylin for 5 min and then rinsed in running tap water for 1 min. Then, the sections were stained in eosin for 30 s, dehydrated, and mounted using general methods. Images were taken using a Nikon Intensilight Fluorescence Microscope (Nikon, Tokyo, Japan).

1.8. Immunohistochemistry (IHC)

Immunohistochemistry (IHC) was performed to analyze the Ki-67 expression in the lung and tumor to confirm the 4T1 tumor metastasis. Briefly, formalin-fixed, paraffin-embedded tumor and lung tissues were sectioned at 5 µm thickness, mounted on charged glass slides, and baked at 60 °C for 1 hour. Sections were deparaffinized in xylene and rehydrated through a graded ethanol series to distilled water. Antigen retrieval was performed using citrate buffer (10 mM, pH 6.0) in a pressure cooker for 15 minutes, followed by cooling to room temperature. Endogenous peroxidase activity was quenched by incubating the slides in 3% hydrogen peroxide for 10 minutes. Slides were then washed in PBS and incubated in SignalStain® Boost IHC Detection Reagent (HRP, Rabbit) (Cell Signaling Technology, 8114S) as a blocking reagent for 10 minutes at room temperature. Rabbit monoclonal anti-Ki-67 (clone D3B5, CST #12202T) was applied at 1:400 dilution. Slides were incubated with the primary antibodies overnight at 4 °C in a humidified chamber. After washing, HRP-conjugated secondary detection was achieved using SignalStain® Boost IHC (HRP, Rb) according to the manufacturer's instructions. Chromogenic detection was performed using SignalStain® DAB Substrate Kit (CST #8059P), with color development monitored under a microscope. Sections were counterstained with hematoxylin, dehydrated through graded ethanol and xylene, and mounted with coverslips using a permanent mounting medium. Images were taken using a Nikon Intensilight Fluorescence Microscope (Nikon, Tokyo, Japan).

2. Supporting Figures

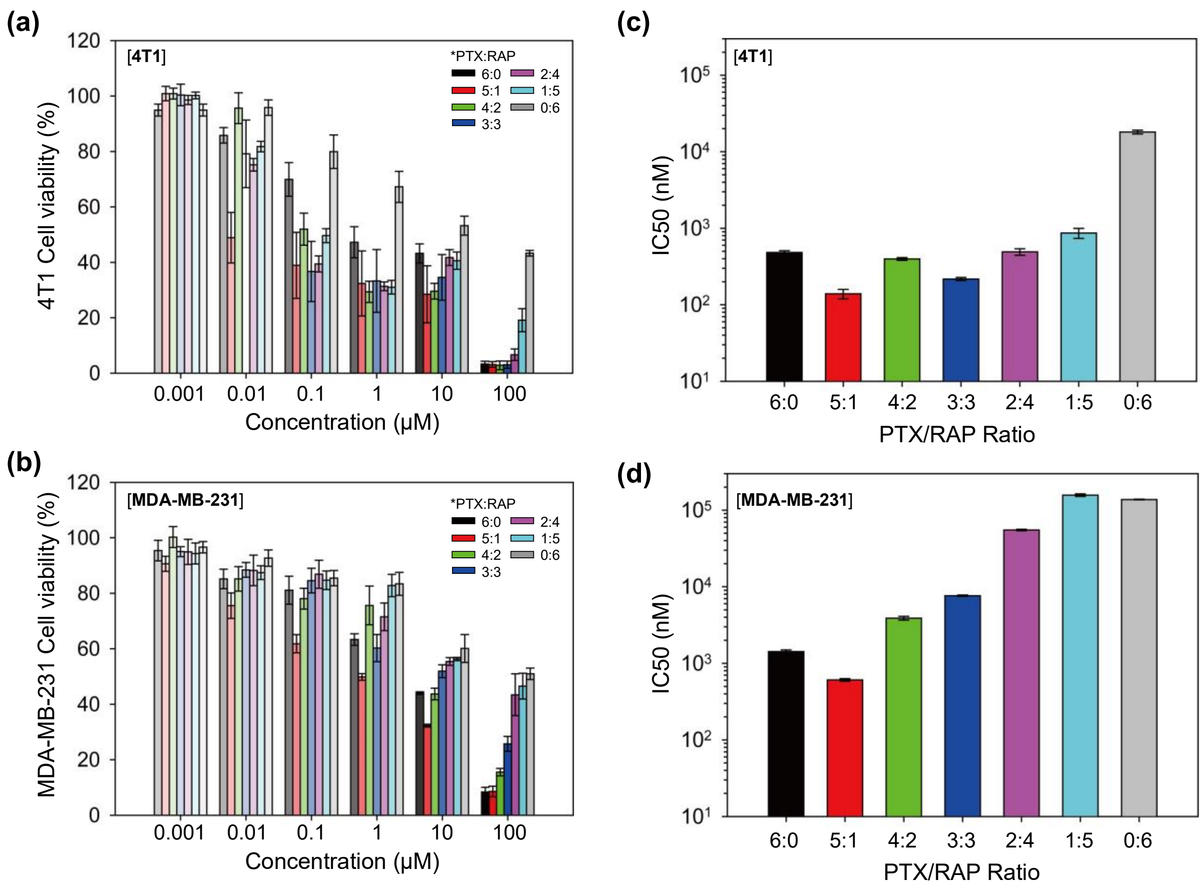

Figure S1. Cytotoxicity of Paclitaxel (PTX), Rapamycin (RAP), and its combination. Cell viability of (a) 4T1 and (b) MDA-MB-231 cells after treatment PTX/RAP combination (6:0 to 0:6) for 72 h at 37 ˚C. Shading corresponds to the concentration. IC50 values for (c) 4T1 cells and (d) MDA-MB-231 cells.

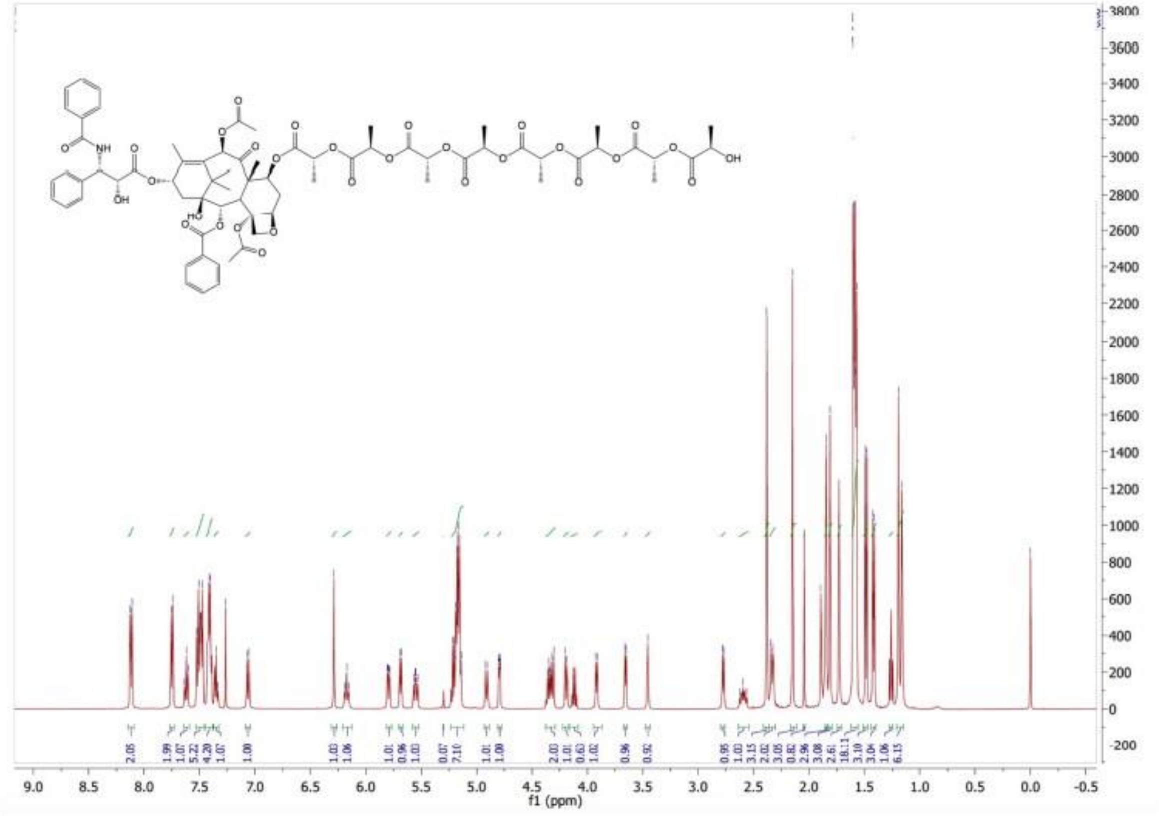

Figure S2. ^{1}H NMR spectra (500 MHz) of oLA_8-PTX in $CDCl_3$.

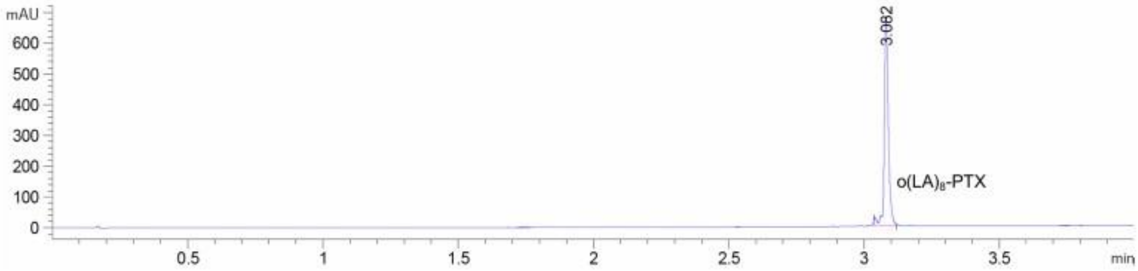

Figure S3. LC-MS spectra of oLA_8-PTX.

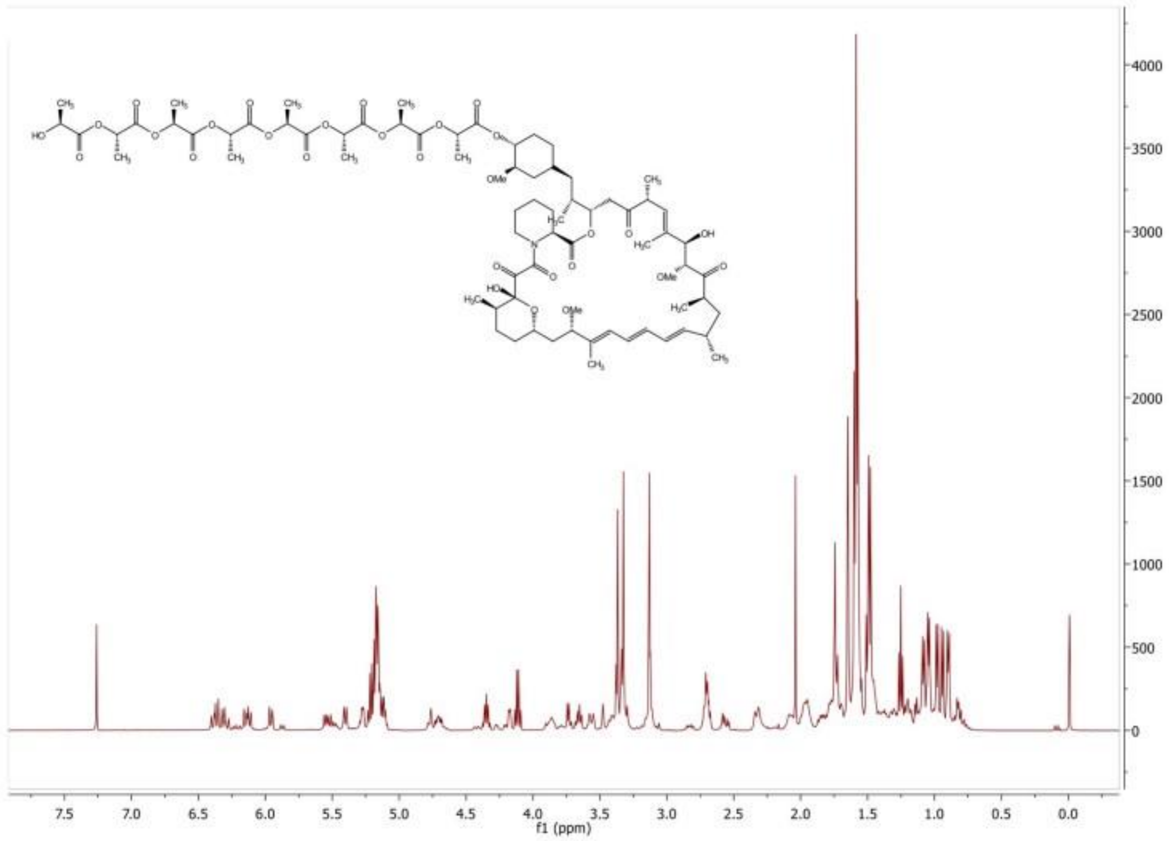

Figure S4. ^{1}H NMR spectra (500 MHz) of oLA$_8$-RAP in $CDCl_3$.

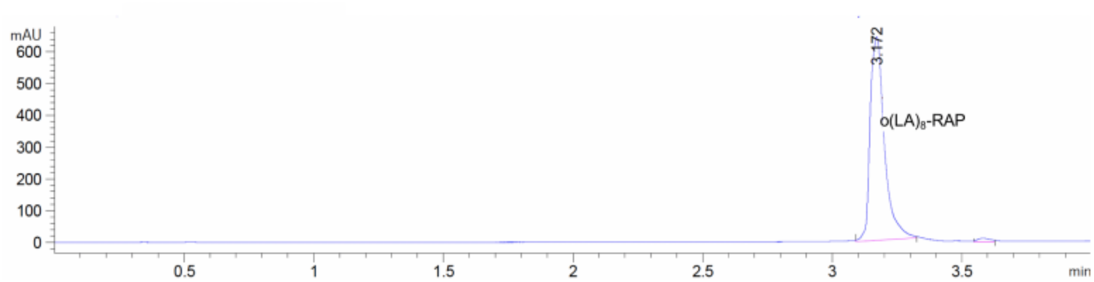

Figure S5. LC-MS spectra of oLA_8-RAP.

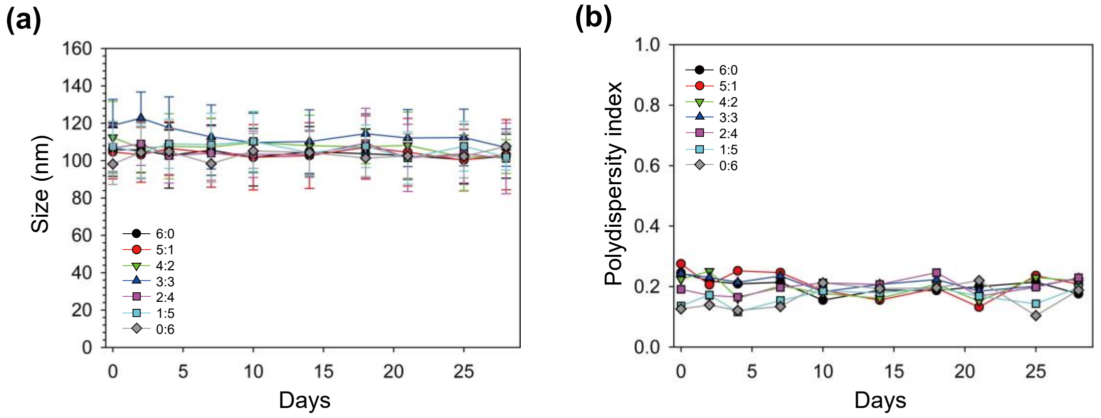

Figure S6. The colloidal stability of micelles in DI water for 28 days. (a) Average size variation of micelles. (b) Changes in polydispersity index (PDI) value of micelles for 28 days.

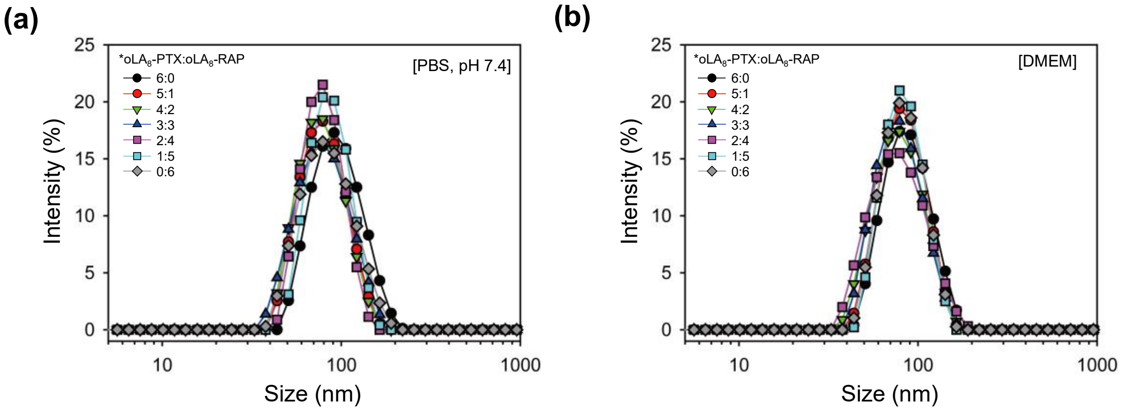

Figure S7. Average hydrodynamic size (intensity distribution) of micelles encapsulating various ratios of oLA_8-PTX/oLA_8-RAP in (a) PBS (pH 7.4) and (b) Dulbecco's Modified Eagle Medium (DMEM).

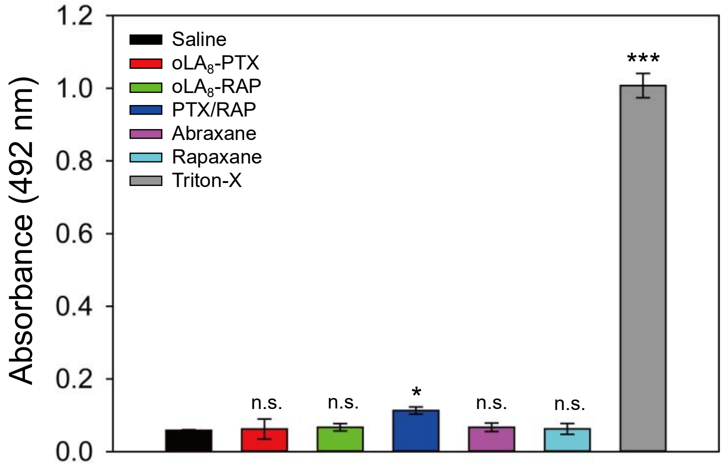

Figure S8. Hemolysis analysis of Rapaxane vs. other treatment groups. Blood samples were incubated with 1x PBS (pH 7.4, negative control), 0.1 mg/mL of oLA_8-PTX loaded micelles (oLA_8-PTX), oLA_8-RAP loaded micelles (oLA_8-RAP), PTX/RAP co-loaded micelles (PTX/RAP), Abraxane®, Rapaxane, and 0.1% (v/v) Triton X-100 (positive control) for 1 h at 37 °C.

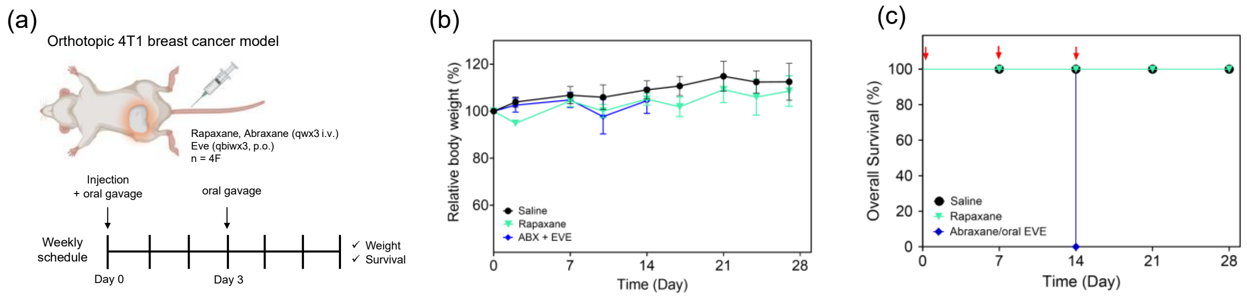

Figure S9. Evaluation of Rapaxane (30 mg/kg PTX-eq) vs Abraxane (30 mg/kg PTX-eq) + oral EVE (6 mg/kg) in the orthotopic breast cancer model. (a) Experimental plan and dosing schedule. (b) Body weight changes. (c) Survival rate.

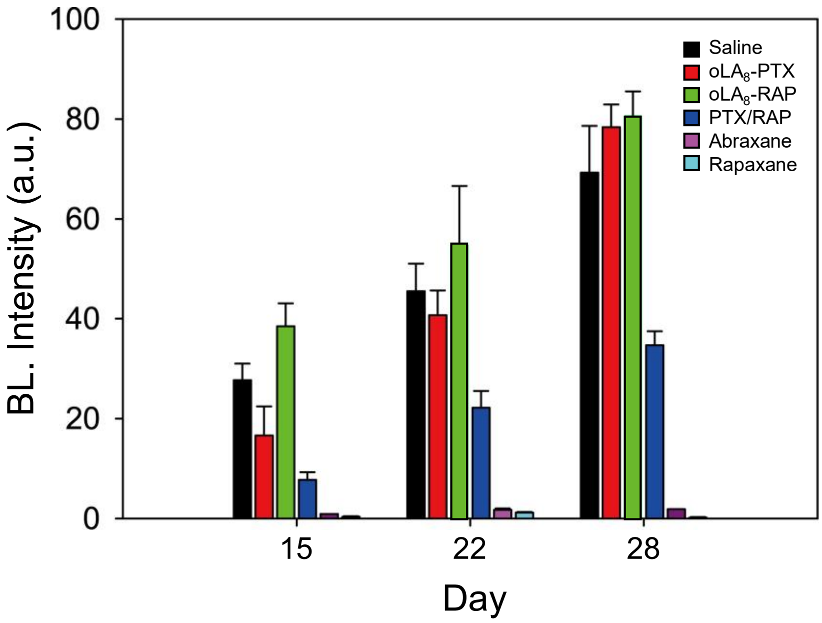

Figure S10. Metastasis to the lung quantified by the intensity of the bioluminescence signal for different treatment groups at various time points.

3. Supporting Tables

Table S1. IC50 values for various ratios of Paclitaxel (PTX) and Rapamycin (RAP) toward 4T1 and MDA-MB-231cells after 72 h incubation at 37 ˚C (n = 8 replicates; mean ± S.D.).

**PTX:RAP ratio**	**4T1**	**MDA-MB-231**
6:0	483 ± 26 nM	1,424 ± 74 nM
5:1	139 ± 20 nM	607 ± 24 nM
4:2	397 ± 17 nM	3,892 ± 223 nM
3:3	217 ± 10 nM	7,633 ± 152 nM
2:4	492 ± 48 nM	55,151 ± 1,294 nM
1:5	867 ± 130 nM	157,237 ± 6,063 nM
0:6	18,147 ± 1,030 nM	137,946 ± 1,446 nM

Table S2. Combination index (CI) values of various PTX:RAP ratios against 4T1 and MDA-MB-231 cells at different fractions affected (F_a).

PTX:RAP ratio	4T1			MDA-MD-231		
	CI at F_a 0.5	CI at F_a 0.75	CI at F_a 0.9	CI at F_a 0.5	CI at F_a 0.75	CI at F_a 0.9
5:1	0.242	0.214	0.190	0.356	0.275	0.212
4:2	0.555	0.378	0.257	1.83	1.40	1.08
3:3	0.230	0.181	0.143	2.71	4.78	8.49
2:4	0.357	0.448	0.564	13.2	66.4	338
1:5	0.339	0.681	1.39	19.4	197	2,054

Table S3. IC50 values for various ratios of oligo(lactic acid)$_8$-paclitaxel (oLA$_8$-PTX) and oligo(lactic acid)$_8$-rapamycin (oLA$_8$-RAP) against 4T1 (murine breast cancer) and MDA-MB-231 (human breast cancer) cell lines after 72 hours of incubation at 37 °C (n = 8 replicates; mean ± S.D.).

oLA$_8$-PTX: oLA$_8$-RAP ratio	4T1	MDA-MB-231
6:0	9,628 ± 719 nM	12,829 ± 2,839 nM
5:1	1,088 ± 53 nM	2,666 ± 144 nM
4:2	4,499 ± 413 nM	5,527 ± 369 nM
3:3	7,103 ± 839 nM	9,227 ± 2,077 nM
2:4	6,503 ± 713 nM	13,108 ± 997 nM
1:5	5,341 ± 245 nM	16,772 ± 1,148 nM
0:6	252,021 ± 36,392 nM	301,964 ± 8,947 nM

Table S4. Combination index (CI) values for various oLA$_8$-PTX: oLA$_8$-RAP ratios against 4T1 and MDA-MB-231 cells at different fractions affected (F_a). F_a = 0 corresponds to 100% cell viability, and F_a = 1 corresponds to 0% viability. CI values were calculated using CompuSyn software based on the principles defined by Chou and Talalay.

**oLA$_8$-PTX: oLA$_8$-RAP ratio**	**4T1**			**MDA-MD-231**		
	CI at F_a 0.5	CI at F_a 0.75	CI at F_a 0.9	CI at F_a 0.5	CI at F_a 0.75	CI at F_a 0.9
5:1	0.095	0.052	0.029	0.175	0.380	0.830
4:2	0.317	0.097	0.032	0.293	0.381	0.500
3:3	0.383	0.201	0.117	0.375	0.136	0.051
2:4	0.242	0.124	0.075	0.370	0.227	0.145
1:5	0.110	0.093	0.100	0.264	0.153	0.096

Table S5. Physicochemical properties of PEG-*b*-PLA micelles containing various ratios of oLA_8-PTX and oLA_8-RAP (n = 3; mean ± S.D.). Micelles were prepared using the PEG-assist method. RP-HPLC was employed to quantify prodrug concentrations, enabling the determination of encapsulation efficiency and weight-loading capacity.

oLA_8-PTX: oLA_8-RAP ratio	Encapsulation efficiency (%)	Weight loading (%)	Total weight loading (%)	Loaded prodrug mole ratio
6:0	oLA_8-PTX: 93.7 ± 4.6<br>oLA_8-RAP: 0	oLA_8-PTX: 48.4 ± 4.4<br>oLA_8-RAP: 0	48.4 ± 4.4	-
5:1	oLA_8-PTX: 94.3 ± 3.6<br>oLA_8-RAP: 95.5 ± 1.2	oLA_8-PTX: 40.4 ± 2.9<br>oLA_8-RAP: 8.2 ± 0.2	48.4 ± 3.1	5.2:1
4:2	oLA_8-PTX: 98.2 ± 2.8<br>oLA_8-RAP: 98.9 ± 3.8	oLA_8-PTX: 33.0 ± 1.8<br>oLA_8-RAP: 16.6 ± 1.2	49.6 ± 3.0	2.1:1
3:3	oLA_8-PTX: 98.1 ± 3.8<br>oLA_8-RAP: 95.2 ± 4.6	oLA_8-PTX: 24.9 ± 1.8<br>oLA_8-RAP: 24.2 ± 2.2	49.1 ± 4.0	1.1:1
2:4	oLA_8-PTX: 96.0 ± 5.5<br>oLA_8-RAP: 96.8 ± 4.8	oLA_8-PTX: 16.3 ± 1.7<br>oLA_8-RAP: 32.8 ± 3.0	49.1 ± 4.8	1:1.9
1:5	oLA_8-PTX: 92.1 ± 3.9<br>oLA_8-RAP: 93.6 ± 3.1	oLA_8-PTX: 7.9 ± 0.6<br>oLA_8-RAP: 40.3 ± 2.5	48.3 ± 3.1	1:4.9
0:6	oLA_8-PTX: 0<br>oLA_8-RAP: 97.9 ± 5.1	oLA_8-PTX: -<br>oLA_8-RAP:49.5 ± 4.9	49.5 ± 4.9	-

Table S6. The average hydrodynamic size of polymeric micelles encapsulating various ratios of oLA_8-PTX and oLA_8-RAP in DI water, PBS (pH 7.4), and cell culture media (DMEM).

oLA_8-PTX: oLA_8-RAP ratio	Size (nm) (PDI) in D.W.	Size (nm) (PDI) in PBS	Size (nm) (PDI) in DMEM
6:0	105.9 ± 42.9 (0.245)	92.5 ± 30.6 (0.143)	89.7 ± 26.0 (0.239)
5:1	104.7 ± 43.3 (0.275)	91.9 ± 23.8 (0.199)	90.6 ± 39.3 (0.195)
4:2	112.4 ± 58.0 (0.224)	90.8 ± 28.0 (0.185)	83.4 ± 31.0 (0.129)
3:3	118.9 ± 41.7 (0.243)	87.2 ± 38.1 (0.152)	82.4 ± 24.9 (0.124)
2:4	106.7 ± 37.6 (0.191)	90.4 ± 20.1 (0.160)	82.4 ± 34.5 (0.175)
1:5	107.3 ± 41.3 (0.136)	98.5 ± 23.7 (0.192)	89.5 ± 22.1 (0.092)
0:6	98.2 ± 32.9 (0.126)	93.0 ± 39.7 (0.132)	84.6 ± 22.8 (0.070)

Table S7. Encapsulation efficiency and loading capacity of PEG-*b*-PLA micelles containing 5:1 ratio of PTX:RAP. Micelles were prepared using the thin film hydration method. RP-HPLC was used to quantify prodrug concentrations to determine encapsulation efficiency and weight-loading. (n = 3; mean ± SD).

PTX:RAP ratio	Encapsulation efficiency (%)	Weight loading (%)	Total weight loading (%)	Loaded drug mole ratio
5:1	PTX: 70.7 ± 2.0<br>RAP: 72.1 ± 4.0	PTX: 29.7 ± 1.0<br>RAP: 5.7 ± 0.3	35.4 ± 0.6	5.2:1

Table S8. Characteristics of reconstituted micelles containing various ratios of $o(LA)_8$-PTX and $o(LA)_8$-RAP. Micelle solutions were lyophilized for 24 hours using a VirTis Advantage Pro benchtop lyophilizer. The freeze-dried cakes were reconstituted at room temperature with water, requiring minimal agitation. Particle size is reported as the z-average measured by dynamic light scattering (DLS). The percentage recovered indicates the amount of the original prodrug retained within the micelles following lyophilization (n = 3; mean ± SD).

oLA_8-PTX: oLA_8-RAP ratio	Reconstituted size (nm) (PDI)	Recovered prodrug (%)	Recovered prodrug mole ratio
6:0	110.6 ± 44.2 (0.143)	oLA_8-PTX: 93.3 ± 1.8<br>oLA_8-RAP: 0	-
5:1	109.9 ± 42.8 (0.201)	oLA_8-PTX: 95.4 ± 3.9<br>oLA_8-RAP: 94.1 ± 0.5	5.0:1
4:2	116.2 ± 43.5 (0.211)	oLA_8-PTX: 93.0 ± 2.0<br>oLA_8-RAP: 96.6 ± 2.1	1.9:1
3:3	119.5 ± 50.9 (0.181)	oLA_8-PTX: 95.9 ± 1.2<br>oLA_8-RAP: 92.2 ± 3.6	1.1:1
2:4	111.7 ± 39.0 (0.141)	oLA_8-PTX: 95.3 ± 1.2<br>oLA_8-RAP: 93.8 ± 3.0	1:2.0
1:5	113.1 ± 41.8 (0.187)	oLA_8-PTX: 95.9 ± 2.6<br>oLA_8-RAP: 91.7 ± 2.5	1:4.9
0:6	108.6 ± 53.9 (0.152)	oLA_8-PTX: -<br>oLA_8-RAP: 94.4 ± 0.6	-

Table S9. IC50 values for polymeric micelles encapsulating various ratios of oLA_8-PTX/oLA_8-RAP (n = 8; mean ± S.D.), tested against 4T1 and MDA-MB-231 cells.

**oLA_8-PTX: oLA_8-RAP ratio**	**4T1**	**MDA-MB-231**
6:0	5,607 ± 1,597 nM	4,813 ± 504 nM
5:1	238 ± 14 nM	1419 ± 79 nM
4:2	286 ± 15 nM	2,568 ± 125 nM
3:3	497 ± 34 nM	3,065 ± 144 nM
2:4	437 ± 35 nM	3,542 ± 417 nM
1:5	523 ± 30 nM	14,500 ± 1,463 nM
0:6	22,479 ± 1,884 nM	151,525 ± 11,631 nM

References

1. Tam, Y.T., J. Gao, and G.S. Kwon, *Oligo (lactic acid) n-paclitaxel prodrugs for poly (ethylene glycol)-block-poly (lactic acid) micelles: loading, release, and backbiting conversion for anticancer activity.* Journal of the American Chemical Society, 2016. **138**(28): p. 8674-8677.
2. Tam, Y.T., et al., *Oligo(Lactic Acid)8-Rapamycin Prodrug-Loaded Poly(Ethylene Glycol)-block-Poly(Lactic Acid) Micelles for Injection.* Pharmaceutical Research, 2019. **36**(5): p. 70.
3. Kang, R.H., N.H. Kim, and D. Kim, *A transformable and biocompatible polymer series using ring-opening polymerization of cyclic silane for more effective transdermal drug delivery.* Chemical Engineering Journal, 2022. **440**: p. 135989.
